# Nucleolar proteomics identifies S100A16 as a key nucleolar protein driving breast cancer metastasis

**DOI:** 10.1038/s41419-025-07963-9

**Published:** 2025-08-22

**Authors:** Brandon J. Metge, Mohamed H. Elbahoty, Amr R. Elhamamsy, Anne E. Popple, Bhavya Papineni, Rajeev S. Samant, Lalita A. Shevde

**Affiliations:** 1 Department of Pathology, University of Alabama at Birmingham, Birmingham, AL USA; 2 Birmingham VA Medical Center, Birmingham, AL USA; 3 O’Neal Comprehensive Cancer Center, University of Alabama at Birmingham, Birmingham, AL USA

**Keywords:** Breast cancer, Metastasis, Organelles

## Abstract

Metastasis is the leading cause of poor clinical outcomes in solid tumors; yet despite recent advances many of the driving factors of metastasis remain poorly understood. Tumor cells that successfully metastasize are subject to numerous stress points from intrinsic and extrinsic factors that the cell must overcome to survive and colonize a secondary site. The nucleolus, the site of ribosome biogenesis, serves as a central hub for sensing and responding to cellular stress and plays a crucial role in this process; furthermore, emerging evidence highlights the potential role of ribosome biogenesis in driving metastasis. To further elucidate the interplay between nucleolar function and metastasis, we performed a comprehensive analysis of nucleolar proteomes from primary and metastatic breast cancer cell lines and identified proteins differentially enriched in the nucleoli of metastatic cells, of which 48 showed statistically significant enrichment. Among these, S100A16 was the most significantly enriched nucleolar protein. Notably, chromatin immunoprecipitation mass spectrometry (ChIP-MS) revealed that S100A16 was associated at rDNA loci with RPA194, the catalytic subunit of RNA Polymerase I, indicating a role in rRNA biosynthesis. Functionally, loss of S100A16 disrupted RNA Polymerase I activation and subsequent rRNA synthesis, reversed epithelial-to-mesenchymal transition, inhibited invasion, and reduced metastatic incidence in animal models of breast cancer. Clinically, elevated S100A16 expression correlated with enrichment of ribosome biogenesis pathways and reduced relapse-free survival in metastatic breast cancer patients. In summary, we identified a critical role for S100A16 as a molecular modulator in the nucleolus that impinges upon breast cancer metastasis.

## Introduction

Metastasis remains one of the biggest challenges for breast cancer. Metastatic progression of tumor cells involves cross-functional teamwork of multiple proteins. These proteins belong to different cellular compartments; however, their activities synergistically influence the manifestation of metastatic attributes.

The nucleolus and ribosome biogenesis are emerging as pivotal influencers of metastasis [1, 2]. It is logically appealing as the nucleolus governs several critical cellular functions, such as cell proliferation, cellular response to stress, and DNA damage repair, which are also the key attributes for a tumor cell to persist and establish metastatic colonization [3]. Nonetheless, the mechanistic underpinnings of the influence of nucleolar activity on metastasis remain less understood.

We conducted an unbiased analysis of metastatic tumors and uncovered that ribosome-related mechanisms are enriched in metastatic tumors, informing that nucleolar activity is likely upregulated in metastatic tumors. The nucleolus is a specialized compartment residing within the nucleus and is formed around the ribosomal DNA (rDNA) repeats on acrocentric chromosomes. Its size and number are cell type and cell state-dependent. The major function of the nucleolus is to produce ribosomes and thus the nucleolus has a direct influence on protein translation [4]. Approximately 1500 proteins have been known to localize to nucleoli. Interestingly, about 90% of these nucleolar proteins are located in other cellular compartments in addition to the nucleolus [5, 6]. Despite the fact that the nucleolus is not membrane-bound, the composition of the nucleolus is closely controlled in a context-dependent manner [3, 7]. The protein contents of the nucleolus are always in a state of flux as cells constantly respond to their needs and their surroundings by modulating protein translation. Several genetic disorders and cancers have been associated with nucleolar proteins [8]. Since the nucleolar proteome is dynamic and varies depending on cell type and states, we hypothesized that clues to metastasis may be evident in the protein contents of the nucleoli.

Informed by the enrichment of nucleolar activity in metastatic tumors, we characterized the nucleolar activity of two isogenic pairs of human breast cancer cell lines contrasting metastatic *versus* non-metastatic. MCF10AT cells are tumorigenic but do not metastasize in animal models, wherein, MCF10CA1acl.1 (MCF10CA) derived from passaging MCF10AT multiple times in immunodeficient mice is highly metastatic [9]. Additionally, we used hormone receptor-positive MCF7 breast cancer cells and their metastatic variant MCF7-5624 cells [10]. In comparison to non-metastatic cells, metastatic breast cancer cells demonstrated elevated ribosomal RNA synthesis. Moreover, our studies presented unique and distinct proteomes in the nucleoli of metastatic versus non-metastatic breast cancer cells. Importantly, we describe a unique role of S100A16 in the nucleolus of breast cancer cells and disruption of protein content in the nucleolus, as evidenced by S100A16 modulation, underscores the importance of the nucleolus in potentiating metastasis in breast cancer.

## Materials and methods

Detailed materials and methods are described fully in Supplemental Methods.

### Ethics approval

All methods were performed in accordance with the relevant guidelines and regulations. All animal studies were conducted in accordance with, and with the approval of, the Institutional Animal Care and Use Committee (IACUC) of University of Alabama at Birmingham (UAB), APN-23022.

### Statistical analyses

All data were expressed as mean ± SEM. Statistical analysis was carried out by GraphPad Prism v10.2.3 (GraphPad Software, Boston, MA). All data were statistically analyzed using Student’s *t*-test (two-tailed), one-way ANOVA with Bonferroni’s multiple comparison test or as detailed in the respective figure legend. All data assume normal distribution with equal variance between groups being compared. Power analysis was used to determine adequate sample size for animal studies. A *p*-value of < 0.05 was deemed significant for all analyses.

## Results

### rRNA biogenesis is elevated in metastatic breast cancer cell lines

**Fig. 1:**
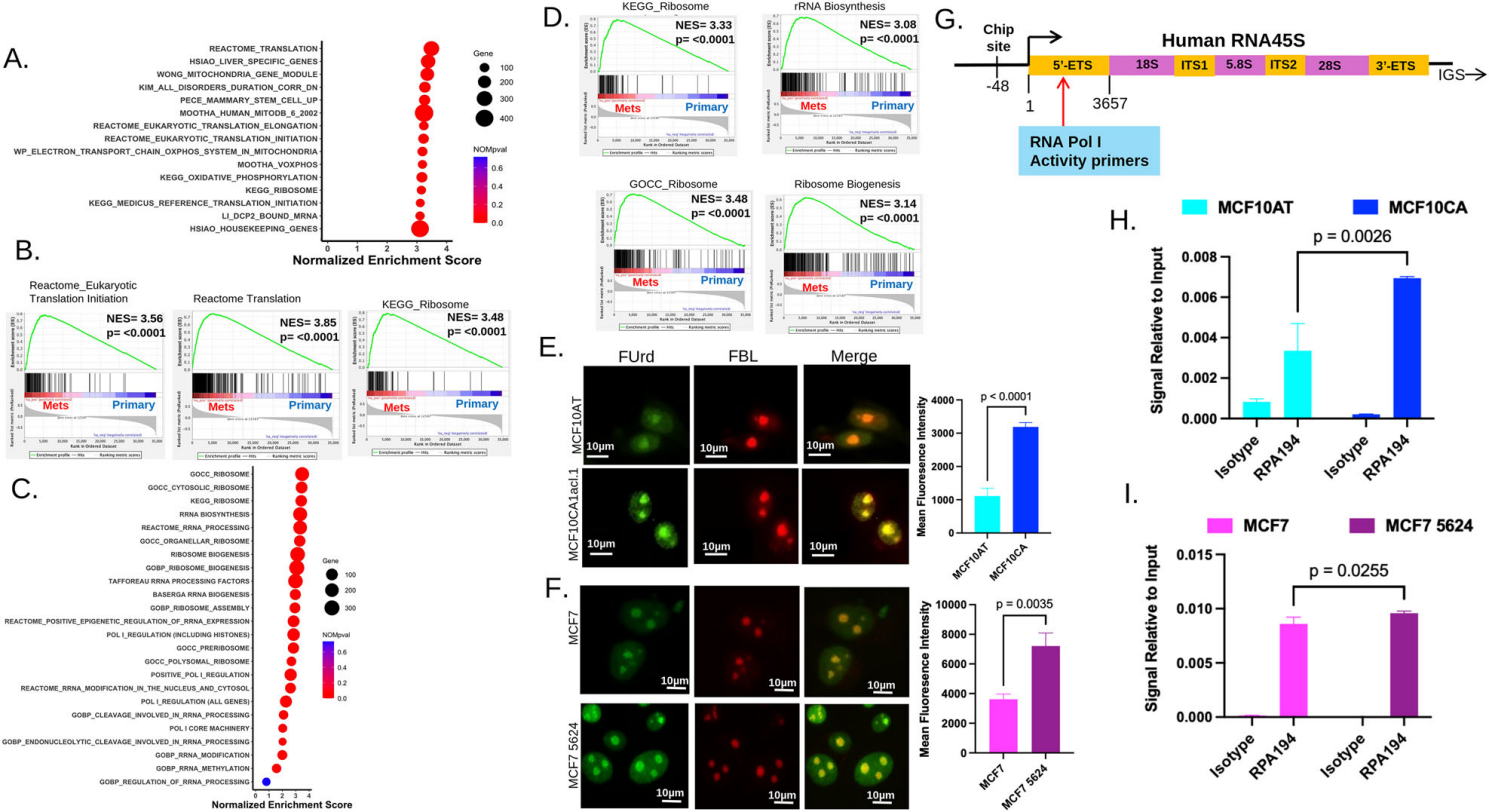
rRNA synthesis is elevated in metastatic breast cancer cell lines. **A** Bubble plot representing gene sets from matched primary versus metastasis from AURORA data set shows the top significantly enriched pathways are related to translation and ribosome. Size of the bubble denotes the number of genes in the gene set with color indicating significance. **B** Enrichment plots depict significant enrichment of translation initiation (NES = 3.56, *p* < 0.0001), translation (NES = 3.85, *p* < 0.0001), and ribosome (NES = 3.48, *p* < 0.0001). **C** Bubble plot representing gene sets from matched primary versus metastasis from AURORA data set specifically shows a significant enrichment of a number of pathways that are related to translation and ribosome. Size of the bubble denotes the number of genes in the gene set with color indicating significance. **D** Enrichment plots depict significant enrichment of KEGG ribosome (NES = 3.33, *p* < 0.0001), rRNA biosynthesis (NES = 3.08, *p* < 0.0001), GOCC ribosome (NES = 3.48, *p* < 0.0001), and ribosome biogenesis (NES = 3.14, *p* < 0.0001). **E** FUrd assay in MCF10CA (metastatic) versus MCF10AT (primary) to measure rRNA synthesis. Representative images depict FUrd incorporation (green) and Fibrillarin (red) overlay. FUrd intensity in the nucleolus was measured across 10 randoms fields and depicted as mean fluorescent intensity. **F** FUrd assay to measure nascent rRNA synthesis in MCF7-5624 (metastatic) versus MCF7 (primary). Representative images depict FUrd incorporation (green) and Fibrillarin (red) overlay. FUrd intensity in the nucleolus was measured across 10 random fields. **G** Schematic of the Human 45S rDNA repeat unit. Primer sites used to query rRNA Pol I activity as determined by the 5′ETS regions are indicated. ChIP sites are denoted at the −48 region in the rDNA promoter element. **H** ChIP quantitative real-time PCR as a measure of RPA194 occupancy at the rDNA promoter element at −48 site normalized to isotype in MCF10CA and **I** MCF7 cell lines. Bar graphs are representative data from two independent experiments. Error bars represent standard error of the mean and *p*-values were calculated using a *t*-test with *p*-values denoted above the graphs.

We sought to determine pathways most enriched in metastases as compared to their matched primary tumor by utilizing the AUROA initiative data set; we performed an unbiased gene set enrichment analysis comparing primary tumor *versus* matched metastasis samples. Interestingly, translation was the topmost enriched pathway along with ribosome and translation initiation and elongation (Fig. 1A, B). Furthermore, a targeted analysis yielded significant enrichment across various pathways related to ribosome biogenesis with the most significant enriched pathways being ribosome and rRNA biosynthesis pathways (Fig. 1C, D). In depth targeted analysis of site-specific metastasis revealed that sites in the lung, brain, lymph node, and bone also showed enrichment of ribosome biogenesis-associated gene sets, with the bone having the most significant enrichment, albeit the sample size was limited (Supplemental Fig. 1A–D). While alterations in nucleolar morphology, activity, and rRNA biogenesis are hallmarks of cancer and correlate to tumor progression and poor prognosis [11], the role of the nucleolus, specifically rRNA biogenesis, in metastasis remains poorly understood. To assess the role of rRNA biogenesis in metastatic breast cancer, we utilized two cell line models. MCF10AT are premalignant human breast epithelial cells which develop benign ductal structures in immunodeficient mice, and the MCF10CA cell line is an isogenic malignant derivative that was obtained by serially transplanting tumor fragments of MCF10AT into athymic mice [9]. Likewise, when xenografted, MCF7 cells form primary tumors which do not metastasize, while an isogenic clonal variant MCF7-5624 metastasizes to bone [10]. rRNA transcription is the rate limiting step in the process of ribosome biogenesis and, therefore a critical measure of nucleolar activity. We analyzed the rate of rRNA transcription as a measure of FUrd incorporation into nascent rRNA after a short pulse. Both metastatic cell lines, as compared to the respective primary tumorigenic cell lines, displayed elevated FUrd incorporation (Fig. 1E, F and Supplemental Fig. 2A). Next, we assessed the abundance of the 5′ETS of rRNA, which has a very short half-life and can serve as a measure of rRNA transcription activity (Fig. 1G). In agreement with the FUrd incorporation assay, the abundance of 5′ETS transcripts were significantly elevated in metastatic cells as compared to the non-metastatic cell lines (Supplemental Fig. 2B, C). rRNA transcription is executed by the multicomponent RNA Polymerase I (RNA Pol I), with RPA194 being the catalytically active subunit. We queried the occupancy of RPA194 at two sites in the rDNA promoter by ChIP analysis (Fig. 1G). This revealed an increased occupancy of RPA194 on the rDNA in both metastatic breast cancer cell lines (Fig. 1H, I). Collectively, these data provide evidence that when compared to their respective non-metastatic counterparts, metastatic breast cancer cells maintain elevated rRNA transcription.

### Metastatic breast cancer cells harbor a unique nucleolar proteome

**Fig. 2:**
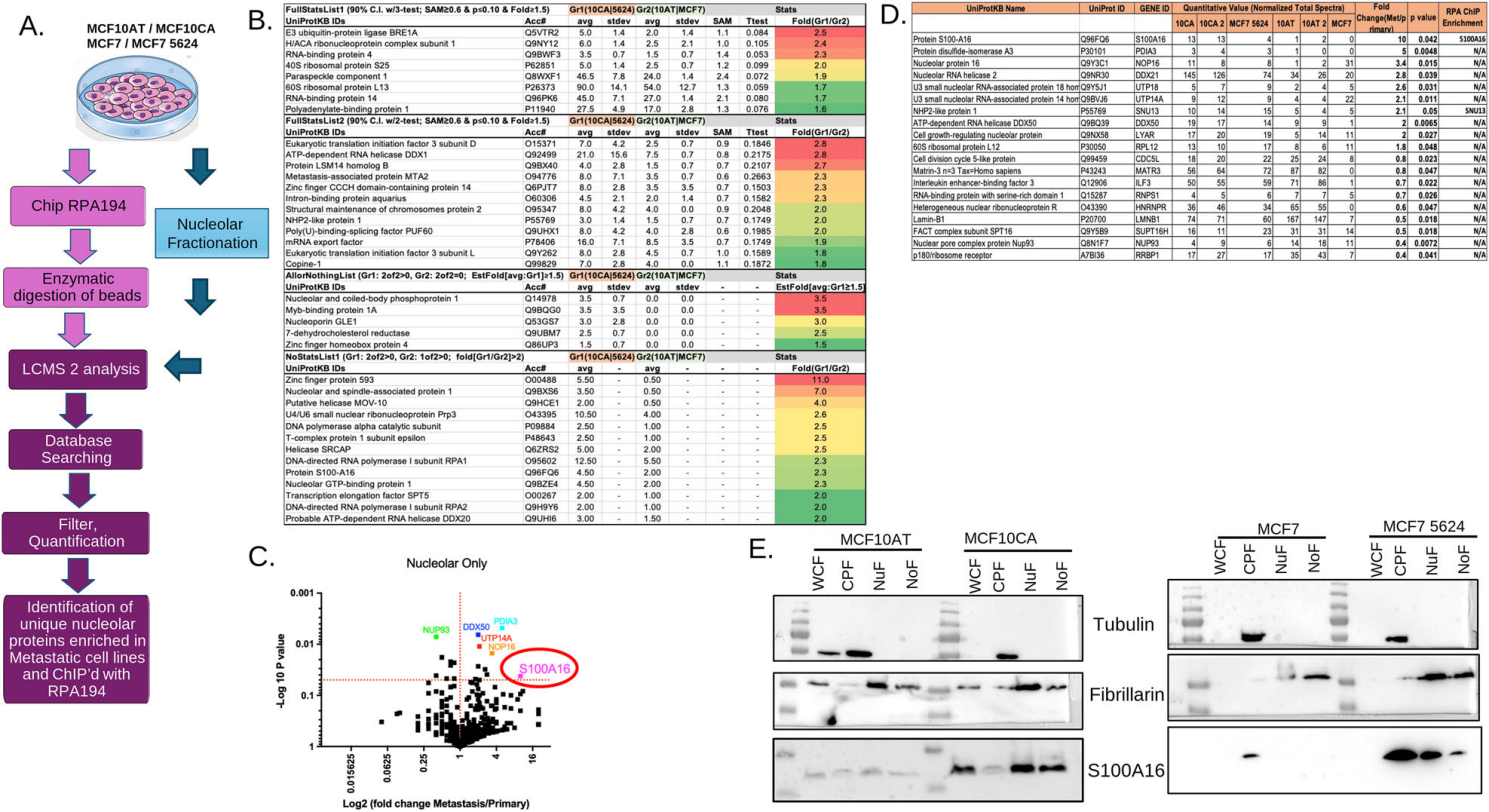
S100A16 is a bona fide nucleolar protein highly expressed in metastatic breast cancer cells. **A** Schematic outline of ChIP mass spec and nucleolar mass spec, which details processing and data analysis post mass spec in both MCF10CA and MCF7 cell lines. A biological repeat of the nucleolar proteome mass spectrometry data was generated from the MCF10 cell line pair. The MCF10 cell line pair was used during the discovery approach and the data was validated using the MCF7 cell line pair, which was run as a single experiment. The ChIP mass spec analysis was carried out in both, MCF7 and MCF10 cell line pairs with each performed as a single run. Data analysis was done by comparing MCF10AT and MCF7 cell lines versus MCF10CA and MCF-5624 cell lines, respectively. **B** Table of top hits from MCF10CA and MCF7 cell lines ChIP’d with RPA194 followed by LCMS analysis to identify proteins bound at the rDNA loci. Data is represented as fold change quantitated from total spectral counts. Each table is representative of top hits from ChIP proteomic data grouped into four categories: (1) proteins detected in all runs across both groups, passing both SAM and one-tailed *t*-tests at ≥90% confidence with fold change ≥1.5; (2) same as (1) but passing only the SAM test; (3) “all-or-nothing” proteins detected in all experimental samples and absent in controls, with average experimental value ≥ 1.5; and (4) proteins found in all experimental samples and only one control, with fold change estimated from group averages (zero values excluded). Group1 (Gr1) MCF10CA and MCF-5624, Group2 (Gr2) MCF10AT and MCF7. **C** Volcano plot of top changing nucleolar proteins from MCF10AT, MCF10CA, MCF7, and MCF7-5624 nucleolar fractions. These proteins have been confirmed as nucleolar from previously published data. **D** Table of nucleolar proteomic analysis, which identified significantly changing proteins in the nucleolar proteomes of MCF10 and MCF7 cell lines. Data is presented as fold change and quantitated from normalized total spectra with *p*-values denoted. Only two nucleolar proteins were noted to be significantly increased in the nucleolar proteomes of the metastatic cell lines that were also identified from the RPA194 ChIP-MS. **E** Western blot analysis of nucleolar fractions identified S100A16 as a bona fide nucleolar protein. Tubulin and fibrillarin western blots validate fractions as cytoplasimic (CPF), nuclear (NuF), or nucleolar (NoF).

The nucleolus is the central site of rRNA transcription with numerous proteins responsible for regulation of nucleolar activity. Therefore, we sought to understand the dynamics of proteins that may be involved in influencing rDNA transcription in the metastatic and non-metastatic cells. We utilized an approach of ChIP followed by LC-MS (ChIP-MS), wherein ChIP was carried out using RPA194 (the major RNA polymerase I subunit), in the metastatic MCF10CA and MCF7-5624 cells and respective non-metastatic MCF10AT and MCF7 cells to identify unique proteins bound at the rDNA loci in association with RPA194 (Fig. 2A). Utilizing Scaffold5 software with total spectral counts we identified a total of 1790 proteins that were associated with RPA194 cumulatively across both cell line pairs, of which 165 were uniquely identified in MCF7 and 79 were unique to the MCF10 cell line pairs demonstrating the overlap in the proteins bound at the rDNA loci in both cell line pairs (Supplemental Table 1). We employed multiple analytical methodologies (as detailed in the “Methods”) to identify proteins that were enriched at the rDNA loci in the metastatic cell lines. The ChIP proteomic data were classified into four categories: (1) proteins consistently detected across all runs in both experimental and control groups, meeting significance in both SAM and one-tailed *t*-tests at ≥90% confidence and showing a fold change ≥1.5; (2) similar to category 1 but passing only the SAM test; (3) proteins present in all experimental samples and entirely absent in controls, with an average experimental intensity ≥1.5 (designated “all-or-nothing”); and (4) proteins detected in all experimental samples and only one control sample, with fold change ≥2 estimated from group averages (excluding zero values). Collectively, these analyses yielded a total of 38 nucleolar proteins (confirmed to be nucleolar from previously published data sets), which were exclusively enriched at the rDNA loci in metastatic cell lines (Fig. 2B) [5, 6, 14]. All in all, the ChIP data provided insight that a small number of nucleolar proteins are preferentially enriched in metastatic breast cancer cells at the rDNA with RPA194, thereby potentially impacting RNA Pol I activity. Given that metastatic cells revealed a unique set of proteins that were enriched with RPA194, we sought to uncover the overall differences in the nucleolar proteome of metastatic versus non-metastatic tumorigenic cell lines. Nucleolar fractions were isolated from MCF10AT, MCF7, MCF10CA, and MCF7-5624 cells and we subjected these fractions to mass spectrometry analysis to identify differentially expressed proteins in the metastatic cells (Fig. 2A). Using Scaffold5 with normalized total spectra we identified a total of 1094 proteins, of which 48 proteins showed statistically significant differential expression in the nucleoli of both metastatic cell lines. Of those 48 proteins, 19 were identified as being bona fide nucleolar proteins as determined from previously published data sets [5, 6, 12]. Furthermore, of the 19 significantly altered nucleolar proteins, only 10 were upregulated in both metastatic lines as compared to the primary cell lines, whereas there were 9 proteins downregulated in both the metastatic lines (Supplemental Table 2). Interestingly, S100A16 presented as the top significantly enriched nucleolar protein in metastatic cells, showing about tenfold increase in the metastatic cell lines as compared to their non-metastatic counterparts (Fig. 2C and Supplemental Fig. 3). Furthermore, when comparing the nucleolar proteins that were significantly enriched in the metastatic cells to the ones that were associated with RPA194 (ChIP-MS) (Fig. 2B), S100A16 was one of only two proteins (other protein was SNU13) that were significantly upregulated and found to be associated with RPA194 at the rDNA loci in metastatic cells. Overall, S100A16 stood out as the topmost hit (Fig. 2D).

S100A16 is a calcium-binding protein which acts as an intracellular calcium sensor and been reported as a potential biomarker in a variety of cancer types as well as reported to influence a variety of molecular mechanisms that promote tumor metastasis [13–15].To further confirm the differential nucleolar localization of S100A16, nucleolar fractions from both primary and metastatic cell line pairs were queried for S100A16 protein expression. In both metastatic cell lines, there was a marked increase in the presence of S100A16 in the nucleolus as compared to the respective non-metastatic counterpart (Fig. 2E and Supplemental File 1). All in all, we identified that metastatic breast cancer cells harbor a unique proteome, which is highlighted by a significant enrichment of S100A16 in the nucleolus.

### S100A16 impinges upon RNA Pol I activity and epithelial mesenchymal transition

**Fig. 3:**
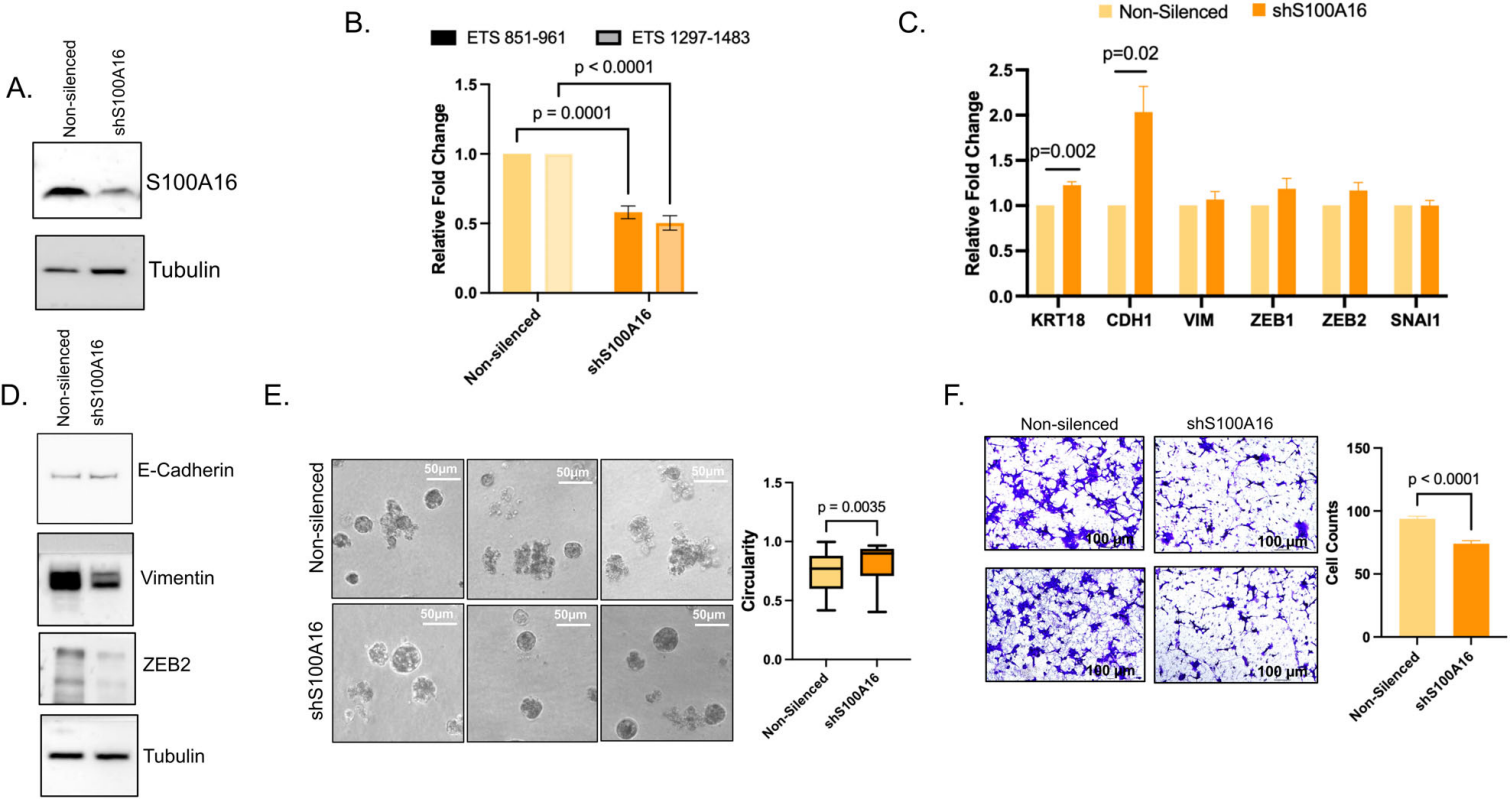
S100A16 is responsible for maintaining an invasive phenotype in metastatic breast cancer cell lines. **A** S100A16 western blot confirms knockdown in MCF10CA stable cell line. Tubulin was used as loading control. **B** Real-time PCR of the 5′ETS as a measure on RNA Pol I activity in cells silenced for S100A16. **C** Real-time PCR of markers of EMT, CDH1, and KRT18 (epithelial markers) and VIM, ZEB1, ZEB2, SNAI (mesenchymal markers) in MCF10CA S100A16 silenced versus control cells. **D** Western blot analysis of EMT markers E-cadherin, Vimentin, ZEB2 in S100A16 silenced MCF10CA1acl.1. Tubulin was used as a loading control. **E** Representative phase images of S100A16 silenced cells grown in a 3D culture assay. Adjacent box plot represents quantification of circular morphology as determined by analysis with Image J software. **F** Representative images of S100A16 silenced MCF10CA invasion assay stained with crystal violet. Adjacent graph depicts counts across 30 random fields of cells invaded.

Next, we sought to unravel the functional role of S100A16 in the nucleolus. To this end, using MCF10CA as a model system, we silenced S100A16 (Fig. 3A and Supplemental File 1). Given that the principal role of the nucleolus is transcription and processing of rRNA, we queried these cells for RNA Pol I activity as measured by 5′ETS transcript levels. RNA Pol I activity was significantly decreased in S100A16 silenced cells as compared to vector control (Fig. 3B). Previous reports from our group as well as others, have unraveled the complex relationship between ribosome biogenesis and epithelial mesenchymal transition (EMT) [16, 17], while S100A16 is reported to influence EMT [18, 19]. As such, we sought to understand the interplay between S100A16, EMT, and ribosome biogenesis. We assessed the steady state transcript levels and protein expression of hallmark EMT markers in the S100A16 silenced cells. Epithelial markers KRT18 and CDH1 were elevated in silenced cells as compared to controls, with a concomitant decrease in both vimentin and ZEB2 (Fig. 3C, D and Supplemental File 1). Furthermore, MCF7-5624 cells silenced for S100A16 demonstrated a significant reduction in the mesenchymal markers VIM, ZEB1, SNAI1 (Supplemental Fig. 4). The distinct molecular paths adopted by these two cell line pairs for reversion of EMT may be due to the fact that MCF7 cells are more epithelial-like as compared to the MCF10CA cells. Functionally, S100A16 silenced cells failed to form invasive structures in 3D matrix and instead maintained a spheroid shape indicative of an epithelial-like phenotype (Fig. 3E). Following these leads we sought to determine if S100A16 silencing mitigated the cells’ ability to maintain a robust invasive phenotype as determined by their ability to invade through Matrigel; indeed, S100A16 silencing in MCF10CA cells impaired their ability to invade (Fig. 3F).

### Knockdown of S100A16 blunts tumor progression and metastasis

**Fig. 4:**
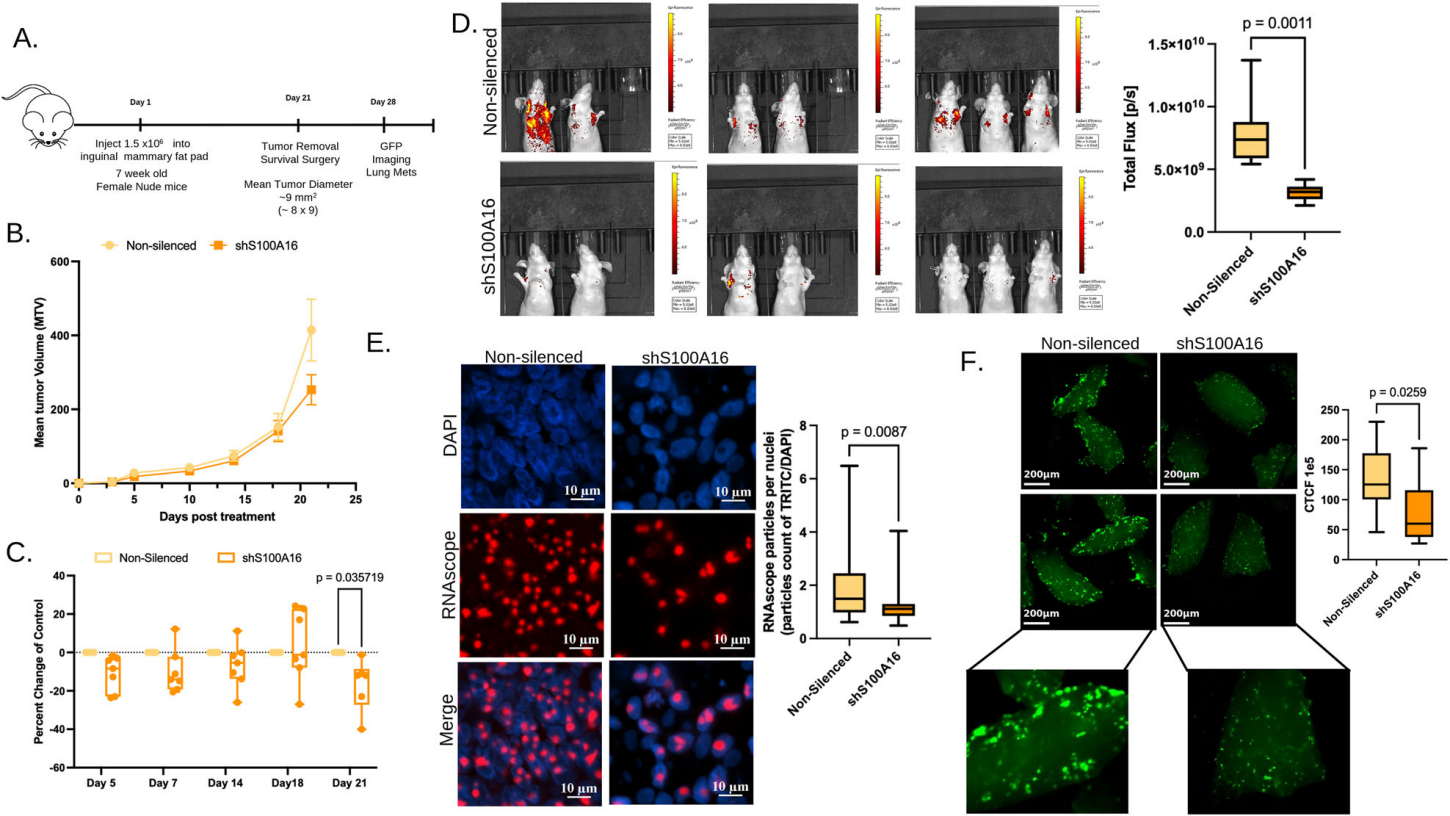
Loss of S100A16 mitigates the metastatic potential of breast cancer cells. **A** Schematic overview of S100A16 silenced MCF10CA tumor model experiment. **B** Mean tumor volume of non-silenced versus S100A16 knockdown MCF10CA plotted as days post injection. Each line is representative of average of 7 mice per group. **C** Waterfall plot depicting percent change in tumor growth of S100A16 silenced cells as compared to non-silenced control. **D** GFP imaging post tumor removal to identify lung metastases in S100A16 silenced and control tumor bearing mice; *n* = 7 mice per group. Quantification of mean GFP intensities using total flux [p/s] from lung metastases. Same ROI selected across each individual. **E** RNAScope of 45S rRNA transcripts in non-silenced versus shS100A16 tumors. Representative images are depicted, DAPI (blue), RNAScope (red). Images were capture across 5 random fields, *n* = 7 for non-silenced *n* = 7 for shS100A16. **F** Representative images from ex vivo PuMA of GFP-labeled MCF10CA S100A16 silenced cells versus non-silenced control. A total of 10 lung sections were generated for each cell line. Graph depicts relative total corrected fluorescence from each lung section. Results are represented as mean +/− SEM *p*-values are denoted.

Our previous set of results revealed that S100A16 impinges upon EMT and drives the invasive potential of breast cancer cells. We postulated that the change in these cellular phenotypes would adversely impact overall tumor progression and metastasis if S100A16 expression is compromised in metastatic breast cancer cells. MCF10CA cells silenced for S100A16 were injected into the fat pads of nude mice and tumor growth was monitored over the course of three weeks (Fig. 4A). At 5 days post injection S100A16 silenced tumors were noticeably smaller than those of the vector control cells; however, minimal differences were seen until 21 days when tumor growth was blunted as compared to controls (Fig. 4B, C and Supplemental Table 3). We next sought to determine the metastatic potential of these tumors given that the overall impedance on tumor growth was modest with the loss of S100A16. Tumors were resected on day 21 and the mice were monitored for metastasis by bioluminescence imaging (Fig. 4A). After 7 days post resection metastases were visible in the lungs of control mice, yet strikingly, most of the S100A16 silenced group had minimal metastases, with a majority of the mice showing no visible metastasis (Fig. 4D). Additionally, we assessed the relative abundance of the 45S rRNA transcript in these tumors utilizing RNAscope as a readout of RNA Pol I activity. S100A16 silenced tumors displayed a significant decrease in the expression of the 45S rRNA transcript as compared to controls; moreover, AgNOR staining revealed a reduced number of nucleoli in the S100A16 silenced tumors (Fig. 4E and Supplemental Fig. 4A). To complement the in vivo assay and evaluate direct lung colonization ability we employed an ex vivo pulmonary metastasis assay (PuMA) with the S100A16 silenced and control MCF10CA cells. Control cells formed large multicellular clusters in the lungs; however, cells silenced for S100A16 were hindered in their ability to form colonies in the lung (Fig. 4F and Supplemental Fig. 4B). These results cumulatively indicate S100A16 alters ribosome biogenesis, which is pivotal in promoting EMT, which ultimately acts to drive the metastatic potential of the cells.

### S100A16 is elevated in patient metastases in correlation with increased ribosome biogenesis

**Fig. 5:**
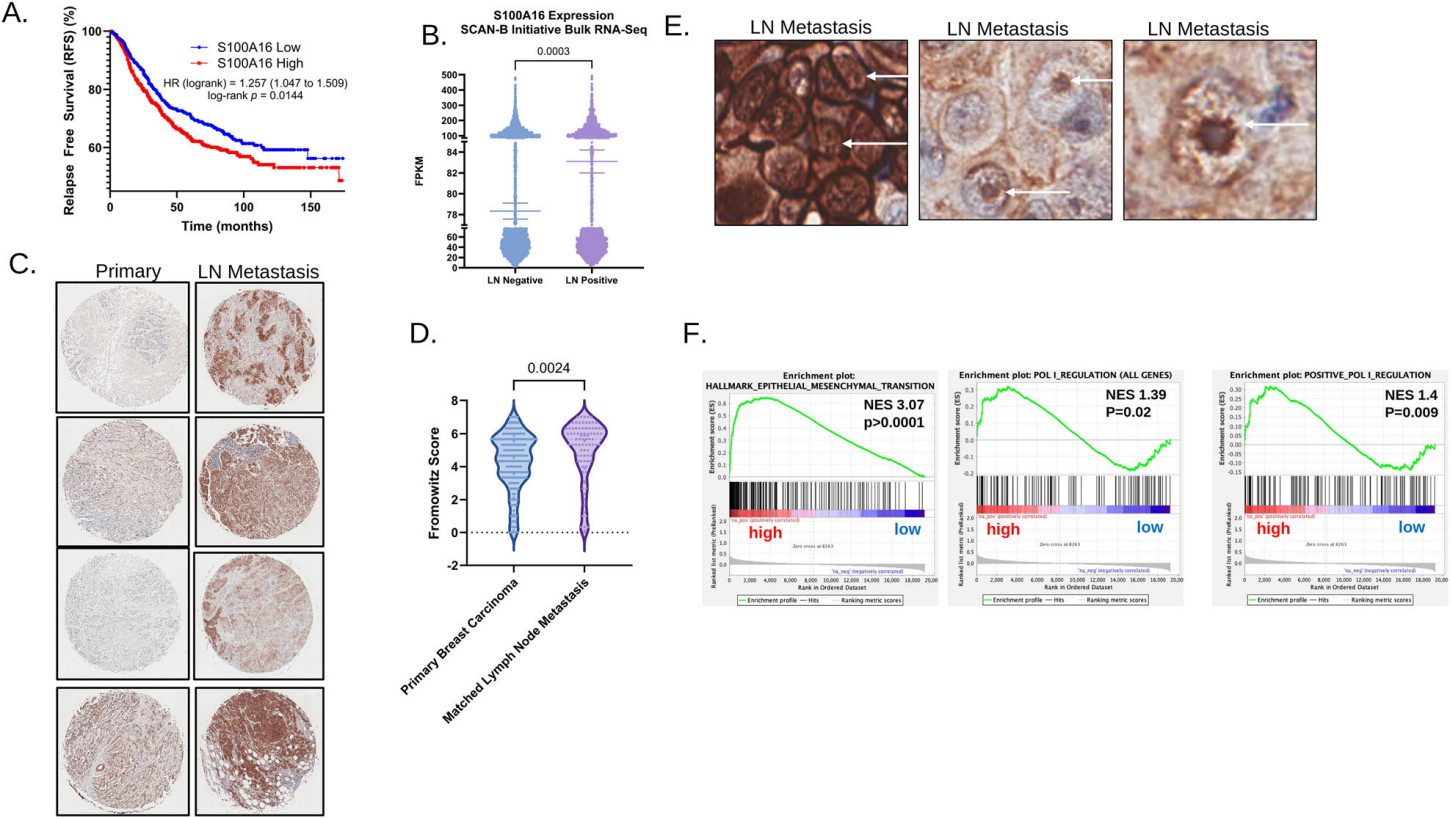
S100A16 is elevated in metastatic breast cancer in combination with increased ribosome biogenesis in patients. **A** Kaplan–Meier curve for relapse free survival (RFS) stratified by tertile 1 and tertile 3 of S100A16 transcript expression of breast cancer patients in GEO, EGA, and TCGA data sets. Log-rank *p*-value was obtained through a Mantel–Cox test. The Hazard Ratio (HR) of 1.257 and significant log-rank *p*-value indicates that high S100A16 expression is a poor prognostic factor for relapse free survival. **B** S100A16 expression from bulk-RNA seq of breast tumors of patients from the Sweden Cancerome Analysis Network–Breast (SCAN-B) initiative data set, parsed by patients with no lymph node metastasis and patients with lymph node metastasis. *T*-test revealed significantly (*p* = 0.0003) increased expression of S100A16 in the tumors of patients who developed lymph node metastasis. **C** Matched primary breast carcinomas and lymph node metastases from tissue microarrays immunohistochemically stained with S100A16 antibody. **D** Fromowitz scoring of five matched metastasis tissue microarrays (BRM961a, BRM961b, BR10010-L87, BR100b, and BR20837a) immunohistochemically stained with S100A16. Each slide was blindly scored three times and the average score was plotted. Overlaps between slides were removed so each case is only represented once. *T*-test between primary scores and lymph node metastasis scores revealed significant (*p* = 0.002) increase in S100A16 protein expression in lymph node metastases as compared to primary tumors. **E** Inset of S100A16 staining in lymph node metastases from (**C**). Arrows depict nucleolar localization of S100A16 in select tumors. **F** S100A16 expression from bulk-RNA seq of breast tumors of patients from the Sweden Cancerome Analysis Network–Breast (SCAN-B) initiative data set was used to stratify patients as either high or low expressing. Enrichment plots depict significant enrichment of Hallmark Epithelial Mesenchymal transition (NES = 3.07, *p* < 0.0001), POLI Regulation (NES = 1.39, *p* = 0.02), and Positive POLI regulation (NES = 1.4, *p* = 0.009) in S100A16 high-expressing patients.

In line with our findings, S100A16 has been previously reported as a potential biomarker, a correlate of poor prognosis, and a driver of metastasis in a variety of cancers [14, 15, 20]. Our query of publicly available datasets for breast cancer revealed that elevated expression of S100A16 in breast cancer correlates with a significantly lower relapse free survival in breast cancer patients (Fig. 5A). To investigate whether S100A16 expression in primary tumors is associated with metastatic events, the Sweden Canceromics Analysis Network–Breast (SCAN-B) dataset was analyzed [21]. Samples were stratified based on lymph node metastasis status (positive or negative), and S100A16 expression levels were assessed using bulk RNA-seq data. Overall, the expression level of S100A16 was significantly higher in those patients with lymph node metastasis (Fig. 5B). Informed by this evidence, we stained biopsy tissue cores from matched primary breast tumor and lymph node metastasis. There was a significant increase in the staining intensity of S100A16 in metastatic specimens, further solidifying the importance of S100A16 in metastatic breast tumor tissue (Fig. 5C, D). Moreover, upon close microscopic examination it was evident that there was defined expression of S100A16 in the nucleolus (Fig. 5E). Given the fact that S100A16 levels indicated overall poor relapse free survival as well as expression of S100A16 was elevated in metastasis, we sought to determine if S100A16 levels also correlated with changes in both EMT and RNA Pol I activation as seen in our previous data. Again, utilizing data from SCAN-B we stratified the patients based on the top and bottom 25% S100A16 expressors and performed targeted GSEA for pathways related to both EMT and RNA Pol I activation. Indeed, when comparing high S100A16 patients to low, there was a significant enrichment of EMT and RNA Pol I gene signatures (Fig. 5F). Collectively, these findings correlate increased expression of S100A16 in breast cancer metastasis with poor prognosis concomitant with the enrichment of pathways related to ribosome biogenesis and EMT.

### S100A16 is elevated during lactation and involution, which mimics the processes during metastasis

**Fig. 6:**
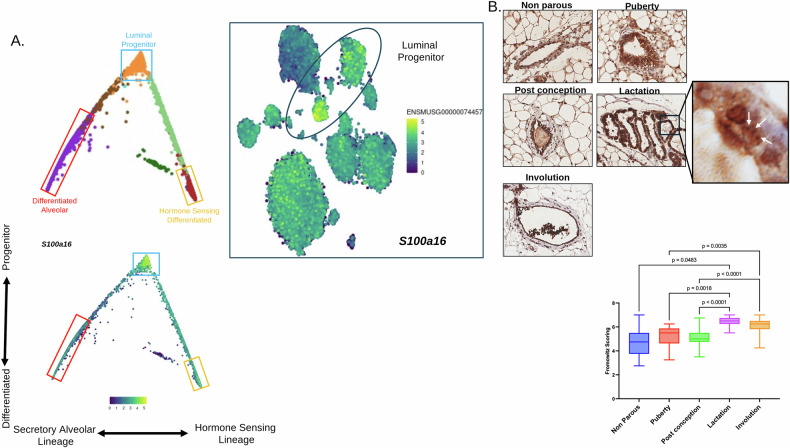
S100A16 is elevated during lactation and involution. **A** Expression of S100A16 in UCSC Mouse Mammary Epithelium scRNA-Seq data set Seurat_v3 clusters. S100A16 expression within the computationally reconstructed luminal compartment derived from single-cell RNA-seq data of mouse mammary cells at various differentiation stages. t-distributed stochastic neighbor embedding (t-SNE) plots showing S100A16 expression across different Luminal cell clusters. **B** Immunohistochemical staining for S100A16 on mouse mammary tissues during different stages of development (puberty, mature non-parous, pregnancy, lactation, and involution. Fromoitz immunoscore for S100A16 staining on mouse mammary tissues during differentiation. Expression was the highest during pregnancy and lactation, then abated during the course of involution. Inset shows representative images during lactation, which demonstrate instances of nucleolar localization as indicated by arrows.

Normal mammary development and its evolution during pregnancy and lactation mimic processes such as EMT that occur during metastatic progression. Remodeling of the mammary gland spanning lactation to involution invokes a series of changes in the microenvironment that can promote tumor cell metastasis [22]. Using xenograft and isogenic tumor transplant models, the involuting mammary gland microenvironment has been shown to support breast cancer tumor growth and metastasis compared to the mammary environment of nulliparous or parous hosts [23]. Notably, the unfavorable prognosis seen in women diagnosed within 10 years after completing a pregnancy is largely attributed to the effects of mammary gland involution [24]. The mammary gland is comprised of multiple cell types, and the mammary ducts are formed by luminal and myoepithelial cells. The mammary gland experiences morphological changes through pregnancy and lactation. We examined single-cell RNA sequencing data spanning different stages of the developing murine mammary gland. S100A16 expression was notably elevated in luminal differentiation (Fig. 6A). Except for the claudin low subtype, the luminal progenitor cells are the credited cell of origin for all other molecular subtypes of breast cancer [25, 26]. Post conception, prior to lactation, mammary ducts aggressively invade through the mammary fat pad for formation of mature-lactating alveoli. Staining of murine mammary tissues at various developmental stages showed that S100A16 expression is highest in lactating mammary tissue and involution (Fig. 6B). Overall, the changes that take place during pregnancy and lactation involve multiple processes that parallel tumor promotion and metastasis. We find that S100A16 expression corresponds with mammary gland developmental stages that mirror key aspects of tumor progression and metastasis.

## Discussion

The importance of ribosome biogenesis in cancer progression has been acutely realized. The physiological demands that a cancer cell withstands warrant a cellular capacity for swift adaptation. This ability creates reliance of cancer cells on effective restructuring of their translational portfolio, which is sometimes warranted in a very short timeframe. Thus, highly dynamic ribosome biogenesis is a fundamental need of a cancer cell, not just for proliferation, but also for maintaining stemness and EMT like phenotype [2, 27]. Several pro-tumor signaling pathways modulate rRNA synthesis leading to its appreciation as one of the hallmarks of cancer [28].

A ribosome is an ensemble of large and small ribosomal proteins (viz. RPLs and RPSs) assembled with rRNA and other ribosome-related proteins. The transcription of rDNA, splicing of rRNA and the assembly of ribosomal subunits almost exclusively occurs in the nucleolus. That functionality requires coexistence of a few thousand proteins within the non-membranous nucleolar domain that exhibits a dynamic flux of its protein contents [4, 6, 12]. Various studies have enumerated nuclear proteomes for a variety of cell types; however, there was a paucity of studies on nucleolar proteomes of metastatic cells. Our efforts were focused on addressing this gap.

Endorsing the leads from unbiased assessment of clinical data, comparison of two distinct isogenic pairs of non-metastatic breast cancer cells with their metastatic counterparts revealed that metastatic breast cancer cell lines maintain an elevated rate of rRNA transcription. Our exploration of the differences in the nucleolar proteomes revealed an interesting perspective. Intersecting the nucleolar proteome with the 38 confirmed nucleolar proteins that associated with RPA194 in the metastatic cells revealed enrichment of only two proteins Viz. S100A16 and SNU13, which highlighted their potential relevance to metastasis. SNU13 (Small Nuclear Ribonucleoprotein 13) is a non-ribosomal protein member of the small subunit processome that is critical for the biogenesis of the 18S rRNA [29]. SNU13 expression is significantly upregulated in Her2-positive breast cancer and displays a negative correlation with prognosis [30]. Published studies that queried publicly available data (GSE2034) related to metastatic breast cancer patients revealed SNU13 as a metastasis-associated gene [31]. These facts certainly make SNU13 interesting; however, in this study, we focused on S100A16 as it was the leading candidate in fold change as well as significance.

S100A16 is a member of the S100 family of calcium-binding proteins. These proteins are characterized by the presence of EF-hand motifs, which enable calcium ion binding and contribute to their structural and functional diversity [13]. S100A16 is widely expressed in various tissues, including the brain, lungs, kidneys, and adipose tissue; yet its expression is often dysregulated in cancer and other diseases [13, 32, 33]. In particular, S100A16 has been identified as a potent biomarker in a number of cancers, such as bladder, gastric, renal cell, pancreatic, lung, and breast cancer, notably that S100A16 has been found as an indicator of poor prognosis [14, 18, 20, 34, 35]. Moreover, our data suggest an important role of S100A16 in metastatic patients, as high levels of S100A16 correlated to an overall decrease in survival. Its expression is often upregulated in tumors, where it contributes to critical hallmarks of cancer, including cell proliferation, invasion, and metastasis [36].

Not only does S100A16 serve as a prognostic indicator across multiple cancer types, but specifically, S100A16 has been shown to be pivotal in tumor cell metastasis, particularly in breast, gastric, renal cell, lung, and pancreatic cancers [32]. Initial reports by Zhou and colleagues demonstrated that S100A16 expression was elevated in breast cancer tissue as compared to adjacent normal. Furthermore, ectopic expression of S100A16 in MCF7 cells led to an increase in cell proliferation and colony formation, but more importantly, both migration and invasion capacity of the cells were enhanced [19]. Moreover, detailed immunohistochemical analysis of S100A16 in breast cancer patients revealed S100A16 as a compelling biomarker which predicted poorer prognosis and was correlated with both tumor size and increased incidence of lymph node metastasis [14]. Molecular studies identified that knockdown of S100A16 in both MCF7 and SKBr3 cell lines suppressed their invasive potential, further demonstrating the importance of S100A16 in potentiating metastatic capacity in breast cancer [14]. Aligned with these reports, our current data emphasizes the ability of S100A16 to modulate EMT and promote the metastatic potential of breast cancer. In addition to breast cancer, S100A16 mediates the invasive potential in a number of other cancer types, namely renal cell, pancreatic, gastric, and lung cancers. S100A16 mRNA and protein levels correlate with overall worse survival and poor prognosis in renal cell carcinoma. Silencing of S100A16 in renal cancer cell lines reduced their migratory potential, facilitated through VEGF and AKT signaling pathways [34]. Multiple groups have elucidated the role of S100A16 in promoting pancreatic cancer metastasis, which in part was shown to be driven via AKT and ERK pathways and impinging upon EMT to drive invasion and migration [15, 37]. Of particular interest, S100A16 expression promoted lung metastasis in pancreatic tumor models; yet, S100A16 knockdown synergized with gemcitabine, significantly reducing pancreatic tumor burden in animal models [37]. Proteomics analysis in gastric cancer identified ZO2, a junction protein known to regulate metastasis, to interact with S100A16. Importantly, S100A16 was shown to promote degradation of ZO2, leading to EMT and enhanced migration and invasion of gastric cancer cells [18]. S100A16 also plays a key role in metastasis of small-cell lung cancer (SCLC) to the brain. Xu et al. demonstrated that S100A16 expression is higher in brain metastases compared to primary SCLC tumors in both humans and mice. Mechanistically, exosomes released from brain endothelial cells were identified as the drivers of S100A16 overexpression in SCLC cells. Thus, brain endothelial cell-derived extracellular vesicles contribute to SCLC brain metastasis by upregulating S100A16 and enhancing cancer cell survival [38]. S100A16 expression is dysregulated in various human cancers, confirming its role in tumor development and metastasis. Except in oral squamous cell and colorectal cancer, where it is not overexpressed, S100A16 is upregulated in several tumor types, promoting increased cell proliferation, invasion, and metastasis through multiple molecular pathways, including PI3K-Akt, MAPK-ERK, JNK/p38, and EMT signaling [32].

The most well-studied cellular location of S100A16 is in the plasma membrane; however, there are independent nucleolar proteome studies that have reported S100A16’s nucleolar location [5, 6]. One such report from Sturchler et al. demonstrated nucleolar localization of S100A16 in mouse glioblastoma and HeLa cells, and upon calcium stimulation, S100A16 translocated to the cytoplasm [13]. Our unbiased analysis of breast cancer nucleolar proteomes revealed that S100A16 is a prominent protein found in the nucleolus of breast cancer cells, specifically those with the highest metastatic potential. Not only was S100A16 found to be located in the nucleolus, but a combined approach of ChIP with mass spec allowed us to discern the location of S100A16 at the rDNA, suggesting a role for S100A16 in regulation in rRNA biosynthesis. Indeed, modulation of S100A16 expression profoundly impacted the regulation of rRNA transcription as well as modulated EMT in metastatic breast cancer cells. Furthermore, across different stages of the mammary gland, S100A16 levels corroborated with dynamic remodeling that occurs during pregnancy and lactation—events that are suggested to be parallel to cellular and molecular changes that occur during tumor progression and metastasis. Multiple studies have identified EMT as the lynchpin by which S100A16 influences many attributes of cancer progression, such as proliferation, migration, and drug resistance. Of note, S100A16 was demonstrated to promote EMT via the Notch pathway in breast cancer as well as direct regulation of ZO2 in gastric cancer [18, 19]. Functionally, S100A16 modulation by gene silencing allowed us to obtain confirmation of relevance of S100A16 in EMT and to the metastatic process. This observation was also endorsed by the increased expression of S100A16 in metastatic specimens and reversion of the EMT phenotype with S100A16 knockdown. We postulate that nucleolar activity of S100A16 promotes EMT in breast cancer cells, driving metastatic potential. Previous reports from our group as well as others, have already provided clues that ribosome biogenesis impinges upon EMT and correlates with changes in invasion and migration [16, 17]. As such, S100A16 may be a pivotal bridge point between ribosome biogenesis and EMT modulation; however, the precise mechanisms by which S100A16 nucleolar function interplays with EMT remain to be fully elucidated.

The nucleolus is a dynamic sub-nuclear body that acutely senses cellular stress and plays an important role in cellular homeostasis [3]. Its prominent role as key player in the regulation of cancer progression as well as metastasis has recently come to light; however, the underlying mechanisms by which nucleolar dynamics regulate these processes remains poorly understood. Our study presented here begins to uncover the complexities of nucleolar activity and the multifaceted role that nucleolar biology plays in cancer progression, in particular metastasis.

Therapeutically targeting nucleolar activity is a lucrative approach given that there are distinct changes in the nucleolus in cancer cells; with our study being the first, highlighting divergent nucleolar proteomes in metastases. Further emphasizing the importance of nucleolar dynamics, a novel inhibitor of metastasis, Metarrestin, was shown to perturb nucleolar structure and inhibit RNA Pol I transcription, thereby accentuating a role for nucleolar activity in driving metastasis [39]. More recently, a study by Ban et. al. has elegantly described a dependence of EMT on ribosome biogenesis, which influences chemoresistance in models of breast cancer [27]. These studies provide further evidence that targeting ribosome biogenesis ultimately impacts EMT, leading to overall changes in invasion, migration, and chemoresistance. Additionally, we provide compelling evidence that the nucleolar proteome has a distinct and unique role in promoting metastasis, as the most differentially expressed protein in the nucleolus of metastatic breast cancer cells, S100A16, is a potent promoter of metastasis. As such, our study provides compelling evidence underscoring the key role the nucleolus plays in metastasis; more specifically, nucleolar S100A16 promotes metastasis by enhancing ribosome biogenesis-driven EMT, representing a potential therapeutic target in metastatic breast cancer.

## Supplementary information

Supplemental Figures

Supplemental Material_Editable file corresponding to the Table depicted in Figure 2B

Supplemental Material_Editable file corresponding to the Table depicted in Figure 2D

Supplemental Figure Legends

Supplemental Methods

Supplemental Table 1

Supplemental Table 2

Supplemental Table 3

## Data Availability

The datasets generated during the current study are available in the Proteomics Identifications Database (PRIDE) repository under the following accession numbers, PXD060651 and PXD060687. The datasets analyzed during the current study are available in the Kaplan Meier Plotter (Kaplan-Meier plotter (kmplot.com), Differentiation dynamics of the developing mammary gland revealed by single-cell RNA-sequencing” database at https://marionilab.cruk.cam.ac.uk/mammaryGland/, Sweden Canceromics Analysis Network–Breast (SCAN-B) data set was downloaded from Gene Expression Omnibus under accession GSE60788, The AURORA data set was downloaded from Gene Expression Omnibus under accession number GSE193103.
