# Supplementary figures and images for: Nucleolar proteomics identifies S100A16 as a key nucleolar protein driving breast cancer metastasis
Source: Cell Death Dis. 2025 Aug 22;16(1):638. doi: 10.1038/s41419-025-07963-9 (PMC12373912; doi:10.1038/s41419-025-07963-9)

A.

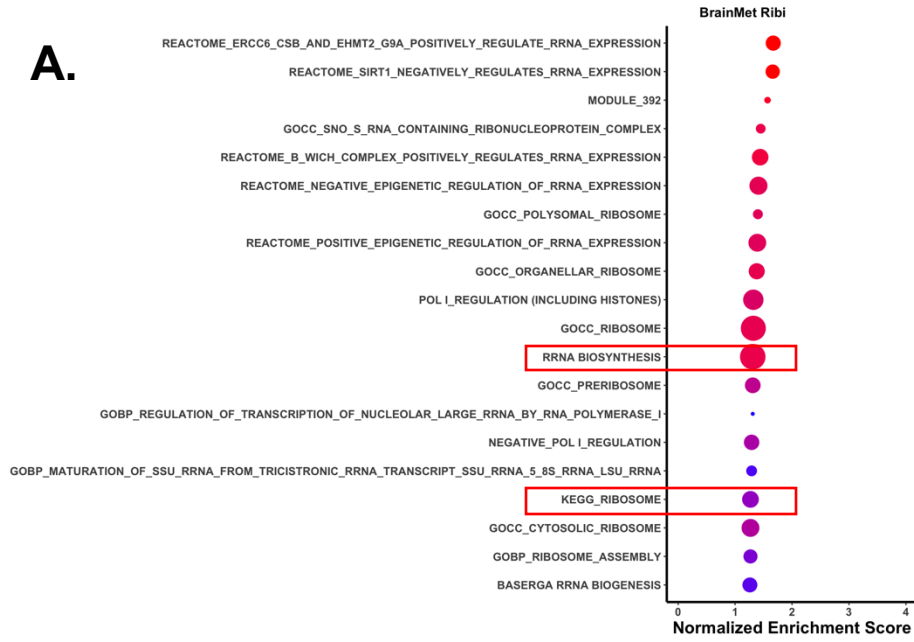

B.

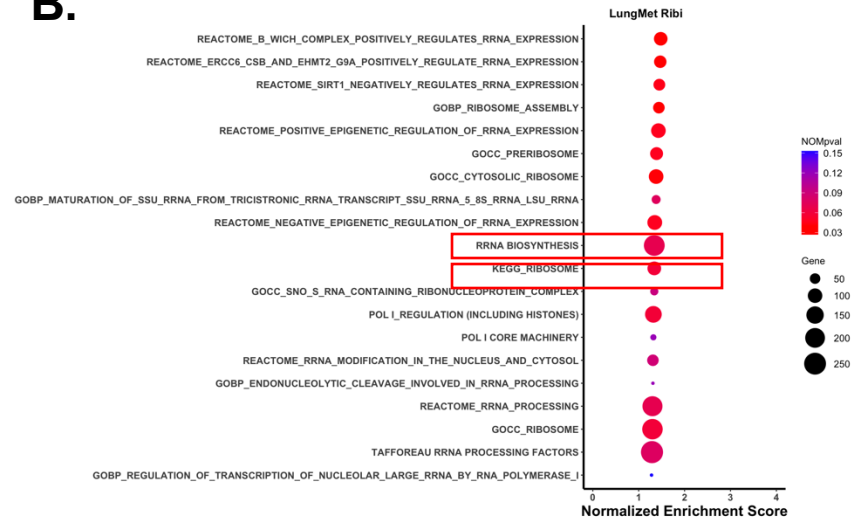

C.

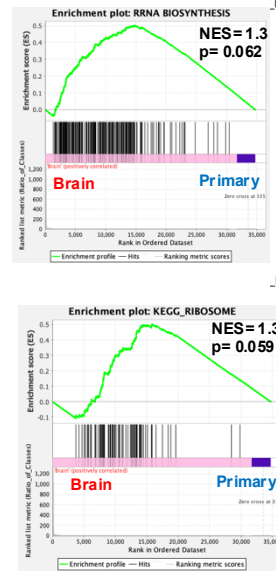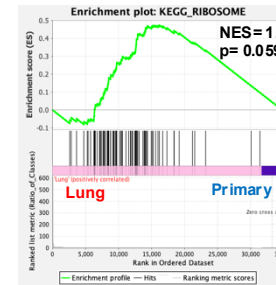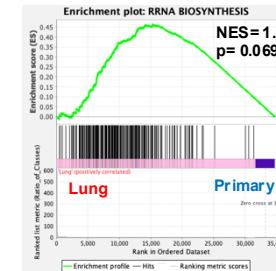

D.

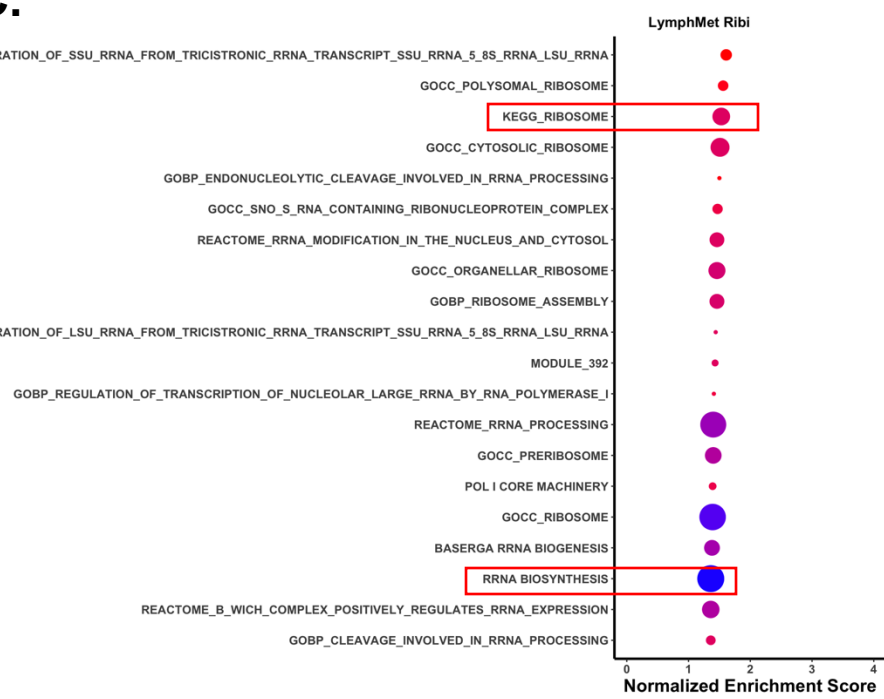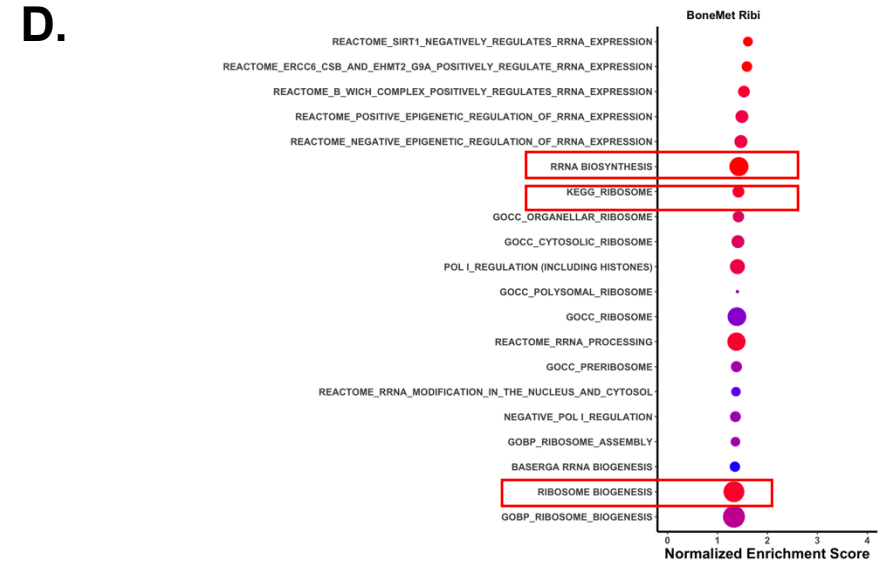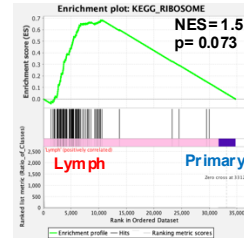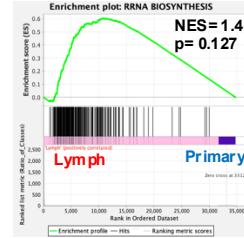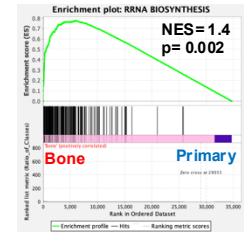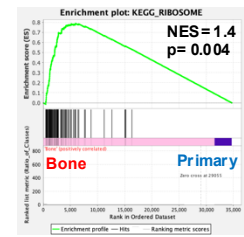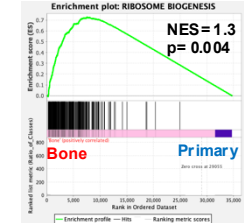

A.

MCF10AT

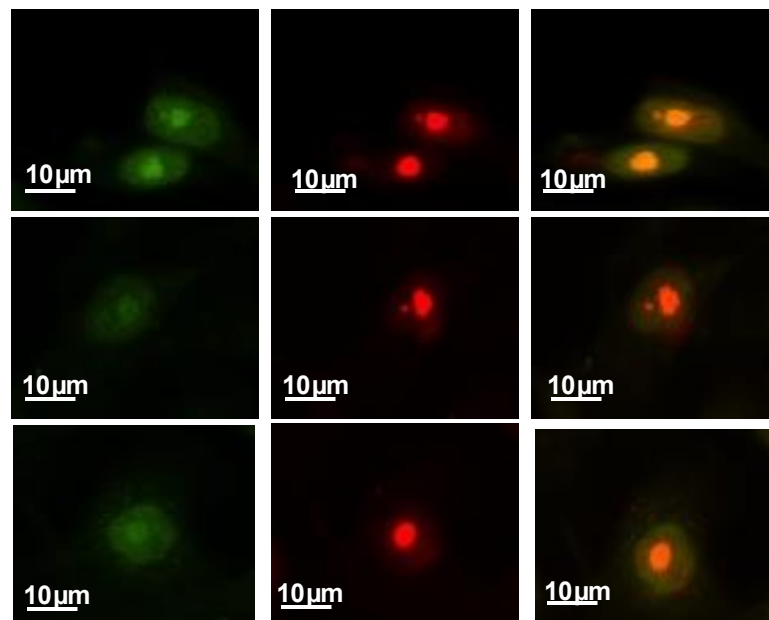

MCF10CA1acl.1

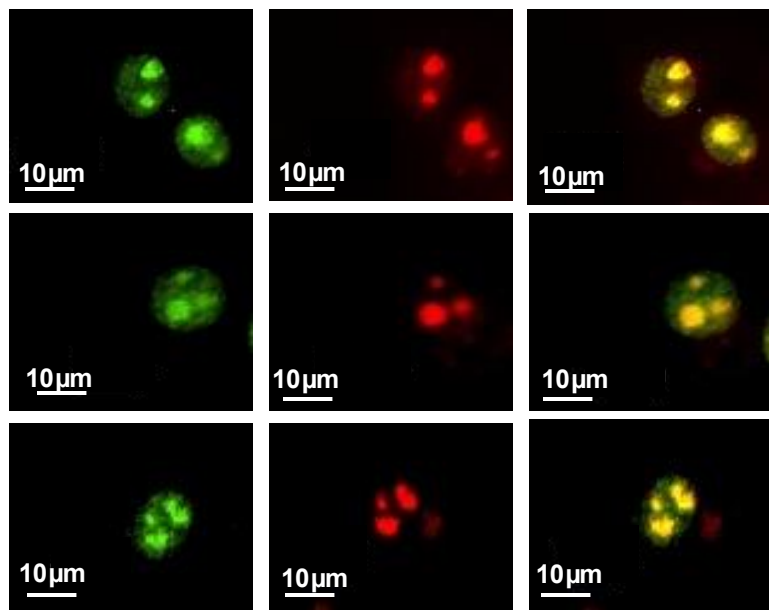

B.

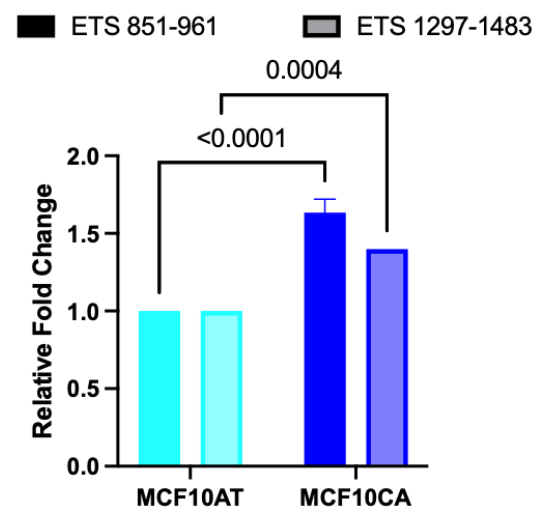

C.

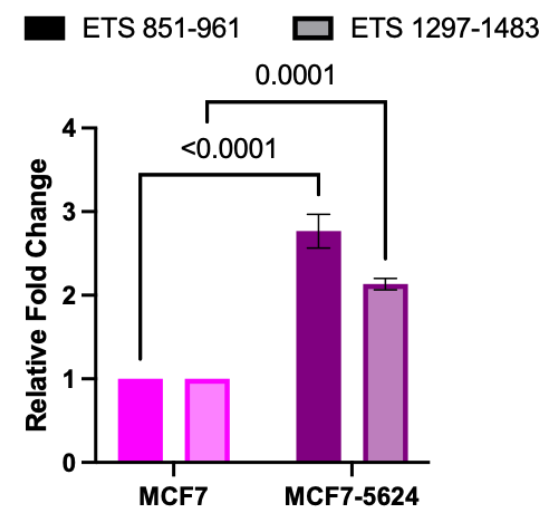

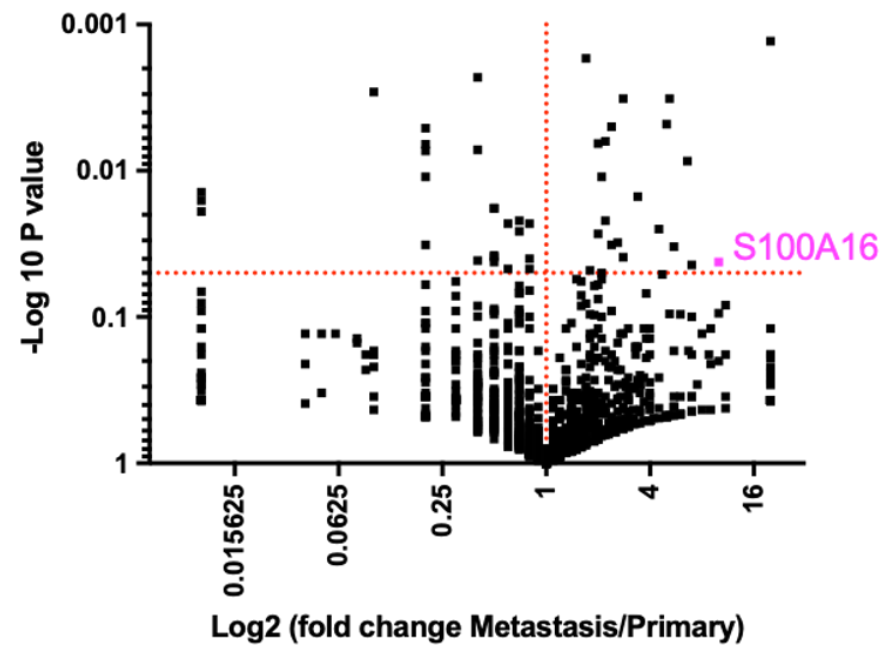

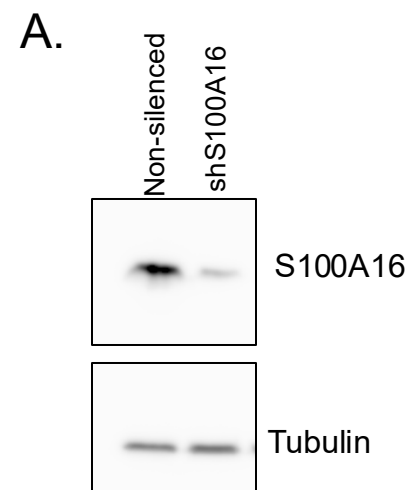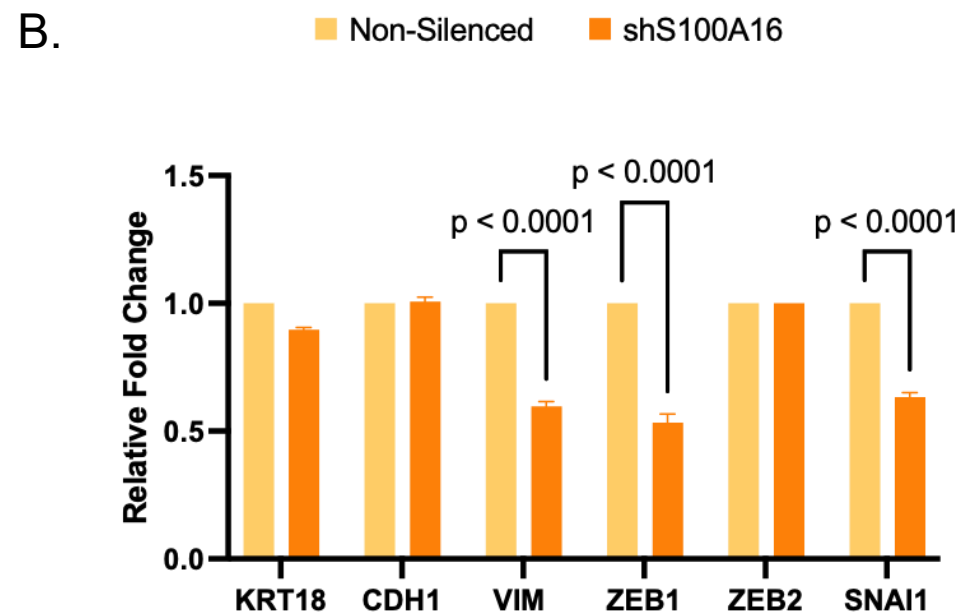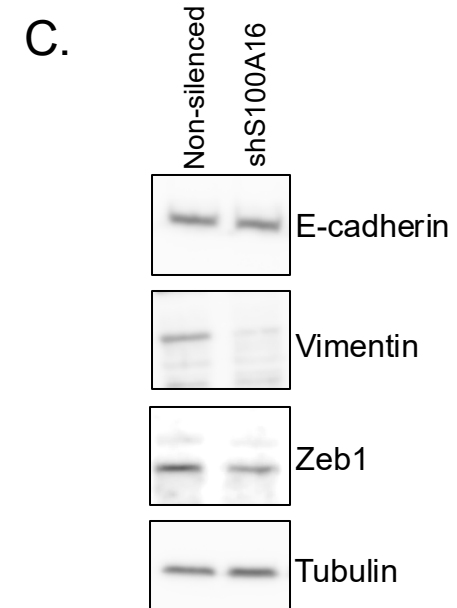

A.

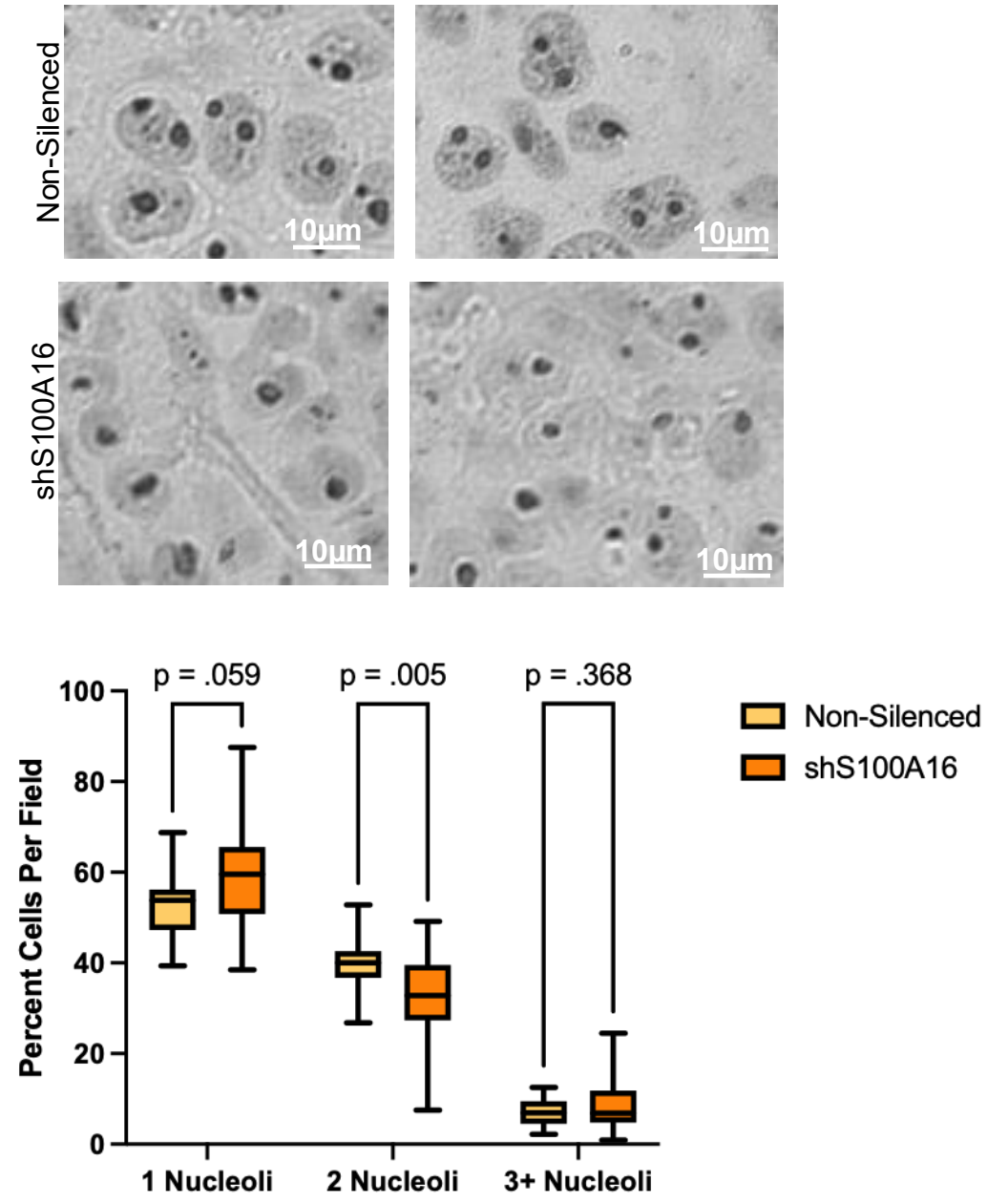

B.

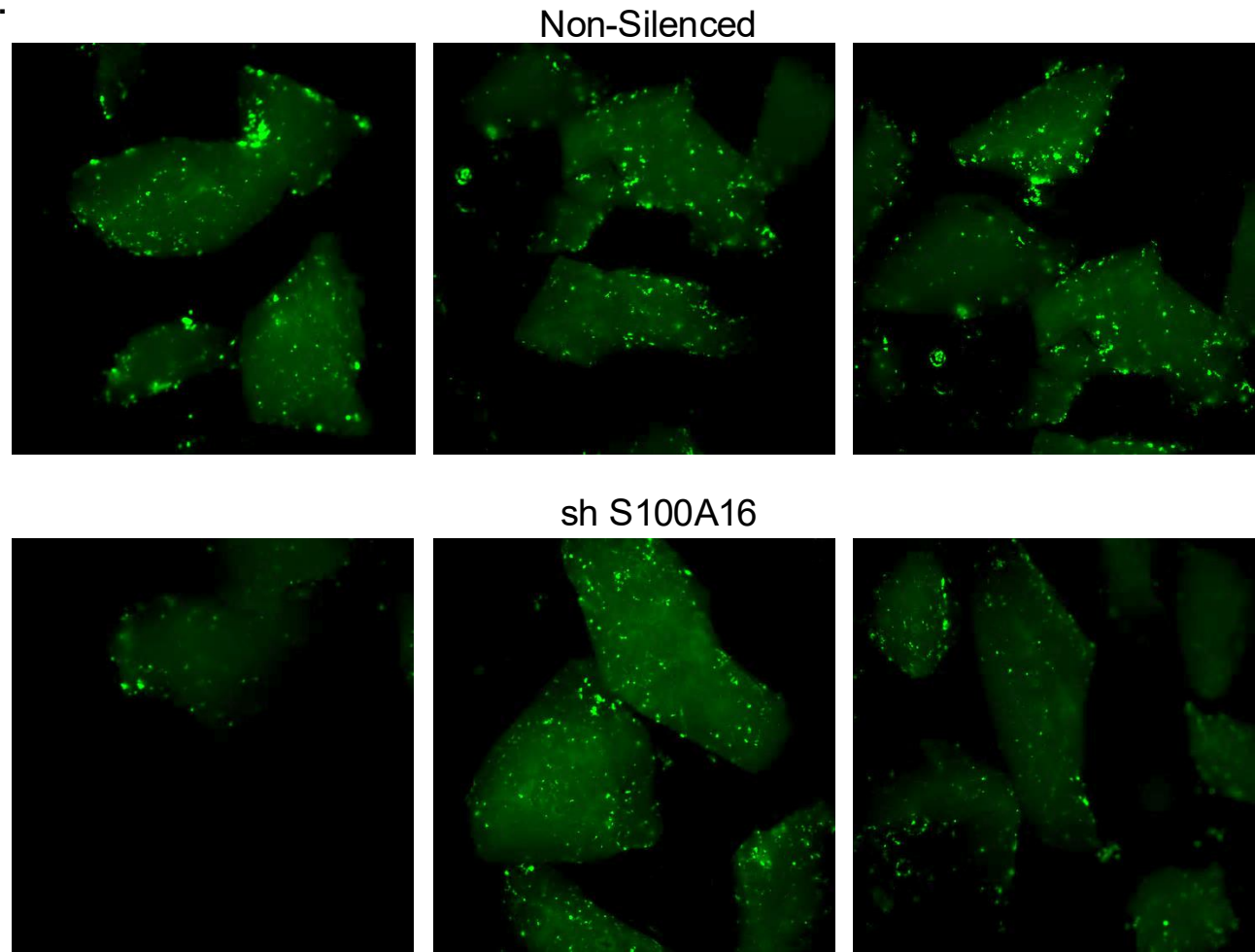

Supplement: Supplementary file 1 — Supplemental Figures [file 41419_2025_7963_MOESM1_ESM.pdf]
