# Supplementary material for: Nucleolar proteomics identifies S100A16 as a key nucleolar protein driving breast cancer metastasis
Source: Cell Death Dis. 2025 Aug 22;16(1):638. doi: 10.1038/s41419-025-07963-9 (PMC12373912; doi:10.1038/s41419-025-07963-9)
Supplement: Supplementary file 4 — Supplemental Figure Legends [file 41419_2025_7963_MOESM4_ESM.docx]

**Supplemental Figure Legends**

**Supplemental Figure 1. (A)** Bubble plot representing of gene sets from matched primary versus brain, **(B)** lung, **(C)** lymph node, or **(D)** bone metastasis from AURORA data set shows the top significantly enriched pathways related to translation and ribosome. Size of the bubble denotes the number of genes in the gene set with color indicating significance. Enrichment plots depict significant enrichment of rRNA biosynthesis (NES =1.4, p=0.002), KEGG ribosome (NES= 1.4, p=0.004), and ribosome biogenesis (NES1.3, p=0.004) only in bone metastasis.

**Supplemental Figure 2.** **(A)** Additional images from FUrd assay of MCF10AT and MCF10CA which depict FUrd incorporation (green) and Fibrillarin (red) overlay. **(B)** Real time quantitative PCR of the 5’ETS as a measure on RNA Pol I activity in the MCF10 cell lines and **(C)** MCF7 cell lines

**Supplemental Figure 3.** **(A)** Expanded volcano plot of top changing proteins from MCF10AT, MCF10CA, MCF7, and MCF7-5624 nucleolar fractions irrespective of being confirmed as nucleolar.

**Supplemental Figure 4. (A)** S100A16 western blot confirms knockdown in MCF7-5624 stable cell line. Tubulin was used as loading control. **(B)** Real-time PCR of markers of EMT, CDH1 and KRT18 (epithelial markers) and VIM, ZEB1, ZEB2, SNAI (mesenchymal markers) in MCF7-5624 S100A16 silenced versus control cells. **(C)** Western blot analysis of EMT markers E-cadherin, Vimentin, ZEB1 in S100A16 silenced MCF7-5624. Tubulin was used as a loading control.

**Supplemental Figure 5.** **(A)** AgNOR staining of MCF10CA1acl.1 shS100A16 (n=5) and non- silenced tumors (n=4). Graph depicts percent of cells per field containing either 1, 2 or 3+ nucleoli per cell. **(B)** Additional representative images from PuMA from different lung sections.
