# Supplementary material for: Nucleolar proteomics identifies S100A16 as a key nucleolar protein driving breast cancer metastasis
Source: Cell Death Dis. 2025 Aug 22;16(1):638. doi: 10.1038/s41419-025-07963-9 (PMC12373912; doi:10.1038/s41419-025-07963-9)
Supplement: Supplementary file 5 — Supplemental Methods [file 41419_2025_7963_MOESM5_ESM.docx]

**Supplemental Methods**

*Cell Culture and Generation of Stable Cell Lines*

MCF10A and MCF10CA1acl.1 cells were grown in DMEM/F-12 (ThermoFisher, Waltham, MA) supplemented with 5% Horse Serum (ThermoFisher), 10μg/ml insulin (Millipore Sigma, Burlington, MA), 25ng/ml hEGF (Millipore Sigma), 250 ng/ml hydrocortisone (Millipore Sigma), and 100ng/ml cholera toxin (Millipore Sigma). MCF7 and MCF7-5624 cells were grown in DMEM/F-12 supplemented with 5% FBS and 10μg/ml insulin. MCF10A and MCF10CA were a kind gift from the lab of Fred Miller. MCF7 was obtained from ATCC, and MCF7-5624 was a kind gift from the lab of Michael Reiss.

MCF10CA1acl.1 and MCF7-5624 S100A16 knockdown cell lines (shS100A16) were generated using lentivirus with GIPZ shS100A16 co-expressing GFP (Horizon Discovery, Waterbeach, UK). Stable cell lines were generated by sorting for GFP positivity and resulting cells were maintained in 500ng/mL puromycin (Millipore Sigma). STR testing was completed for MCF10A, MCF10CA and MCF7, and cell lines were routinely tested for Mycoplasma using Plasmo Test (InvivoGen, San Deigo CA).

*3D Assay for Growth Morphology*

Cells (5 x 10^4^) in growth media supplemented with 2% Cultrex 3D culture matrix (R&D systems, Minneapolis, MN) were seeded onto 8-chambered glass slides (Millipore Sigma) coated with 150µL Cultrex and incubated at 37°C in 5% CO_2_. Media was replaced every 2 days with fresh complete media containing 2% Culturex 3D culture matrix. Images were acquired 4 days after seeding, using a Nikon Eclipse Ti-U microscope (Nikon Instruments Inc., Melville, NY). Image J analysis software was used to measure circularity of spheres.

*rRNA Synthesis Assay*

rRNA synthesis was measured as a readout of FUrd incorporation into nascent rRNA transcripts. Briefly, cells were seeded onto glass coverslips and allowed to attach overnight. Cells were pulsed with 2mM FUrd (Millipore Sigma) for 30 minutes. Following the pulse, cells were immediately fixed with 3.7% formaldehyde for 10 minutes at room temperature. Coverslips were subsequently washed and permeabilized with 0.3% Triton X-100 for 15 minutes and blocked in 5% BSA. Coverslips were incubated with anti-Brdu (Millipore Sigma) and anti-Fibrillarin (Abcam, Cambridge, UK) overnight at 4°C, then incubated with appropriate Alexa Fluor 488 or Alexa Fluor 594 secondary antibodies (Thermo Fisher) and mounted using Vectashield Plus with DAPI (Vector Labs, Newark, CA).

Images were taken with a Nikon Eclipse Ti-U (Nikon Instruments Inc.) using the same exposure times for all images acquired. Mean Fluorescence Intensity was determined using NIS Elements Advanced Research software analyzing cells across 6 random fields. Representative images are depicted.

*Chromatin Immunoprecipitation (ChIP)*

Cells (2.5 x 10^6^) were plated in 10cm petridishes. The subsequent day cells were processed using the Simple Chip Plus Enzymatic kit (Cell Signaling, Danvers, MA) as per manufacturer’s protocol. Briefly, cells were fixed with 1% formaldehyde at room temperature and cell pellets were processed for nuclei isolation and chromatin digestion with Micrococcal Nuclease and sonication. 5 μg of cross-linked chromatin was immunoprecipitated with anti-RPA194 (Santa Cruz Biotechnology, Dallas, TX). Chromatin was then eluted from the IP, and cross-links were reversed. Purified DNA from ChIP and input was subjected to RT-qPCR using 2X Maxima SYBR Green Master Mix (ThermoFisher) along with primer pairs to amplify regions of the rDNA repeat. Primer pairs used were as follows: Promoter -48 For- GAGGTATATCTTTCGCTCCGAGTC Rev- CAGCAATAACCCGGCGG.

Threshold cycle (C[T]) values of input DNA were used to calculate percent input of immunoprecipitation utilizing the following calculation: Percent Input = 2% x 2^(C[T] 2% Input Sample-C[T] IP Sample)^, and the values for the corresponding isotypes were subtracted from the specified antibody used for ChIP. Each reaction was done in triplicate using an Applied Biosystems Step One Plus (ThermoFisher).

*Chromatin Immunoprecipitation Mass Spectrometry (ChIP MS)*

MCF10CA1acl.1, MCF10AT, MCF7 and MCF7-5624 cells (2.5 x 10^6^) were seeded and allowed to attach 24 hours and then processed for ChIP. Briefly, at time of collection, cells were crosslinked using 1% formaldehyde for 10 minutes. Crosslinked cells were washed twice and processed for nuclei isolation and chromatin digestion with micrococcal nuclease followed by sonication. 100µl of protein G beads were washed in 1ml of 1X PBS+ 5mg/ml BSA for a total of 4 washes. 3µg of anti-RPA194 (Santa Cruz) was coupled to washed protein G beads and the mixture was mixed by rotating the tube overnight at 4°C. Antibody coupled beads were washed 4 times in 1X PBS containing 5mg/ml BSA, and incubated with 10μg of crosslinked chromatin overnight at 4°C. Following incubation with chromatin, beads were washed and processed for analysis via LCMS upon processing the beads as follows: Samples were eluted in 1X final LDS sample buffer at 96° C for 10min. The eluate was collected on a magnetic stand, reduced, and denatured further at 70°C for 10min. The sample was resolved using 10% Bis-tris gel and stained overnight with Colloidal Coomassie. Each sample lane was digested with trypsin overnight in 6 fractions prior to LCMS analysis. Data was analyzed using Scaffold 5 with the following Scaffold Proteomics Settings:

Protein Grouping Strategy: Experiment-wide grouping with binary peptide-protein weights

Peptide Thresholds: 80.0% minimum

Protein Thresholds: 99.0% minimum and 2 peptides minimum

Peptide FDR: 2.6% (Prophet)

Protein FDR: 0.0% (Prophet)

For the ChIP proteomic data, we characterized the analysis into four categories as described: 1) significant protein IDs were observed for every run across both groups, here the data had to pass both stats tests (SAM & Ttest[single tail]) at ≥90% C.I. with a set fold change minimum of ≥1.5; 2) proteins were generated in the same fashion as section #1 with the difference of having to pass only the SAM test at ≥90% C.I. and with the same fold change; 3) significant protein IDs were generated for every run across the experimental group with all zero values for the samples in the control group, thereby generating an “all or nothing” list of proteins with an average experimental value of ≥ 1.5; 4) this is the “no-stats” list generated from proteins observed in all samples within the experimental group and only one the control group with an estimated fold change based on averages of experimental vs. control groups (zero values were not used in the averages for any group). When relevant, the individual stats included pseudo-non-parametric statistical analyses performed between each pair-wise comparison. These analyses include 1) the calculation of weight values by significance analysis of microarray (SAM; cut off >|0.6| combined with, 2) T-test (single tail, unequal variance, cut off of p < 0.10), which were then sorted according to the highest fold change for each comparison. For SAM, the weight value (W) is a statistically derived function that approaches significance as the distance between the means (μ1-μ2) for each group increases, and the SD (δ1-δ2) decreases using the formula, W=(μ1-μ2)/( δ1-δ2). For relative protein abundance ratios determined using total spectral counts (TSC’s), we set a 1.5-2.0 fold change (depending on the application) as the threshold for significance, determined empirically by analyzing the inner-quartile data from the control experiments using ln-ln plots, where the Pierson’s correlation coefficient (R) is 0.98, and >99% of the TSCs that fell between the set fold change. In each case, all three tests (SAM, and/ or Ttest [when relevant] & Fold change) had to pass in order to be considered significant.

The ChIP mass spectrometry proteomics data have been deposited to the ProteomeXchange Consortium via the PRIDE partner repository with the dataset identifier PXD060651.

*Nucleolar Fractionation*

Nucleoli were isolated from the cells as previously described. Briefly, the cells were washed and collected in a minimal volume of 1xPBS. They were subjected to osmotic shock, then lysed in NP-40-containing buffer before being homogenized using a tight Dounce homogenizer. Nuclei were purified through a 250mM sucrose cushion, then sonicated to release the nucleoli. Nucleoli were purified through a 340mM sucrose cushion, then resuspended in either in 1X RIPA buffer for western blot or 1xPBS for downstream Mass Spec analysis.

For Mass Spec analysis of the nucleolar fractions, the sample was lysed in 1X LDS sample buffer, sonicated, and an equal amount of protein was resolved using 10% Bis-tris gel and stained overnight with Colloidal Coomassie. Each sample lane was digested with trypsin overnight in 6 fractions prior to LCMS analysis. Data was analyzed using Scaffold 5 with the following parameters: for more stringent analysis, protein threshold was set to 99%, minimum peptides 3, and peptide threshold 80%. To reveal additional hits, moderately stringent analysis was done with 80% protein threshold and peptide minimum set to 1. Quantitative Value (Normalized Total Spectra) was used to determine fold change >2 in protein abundance and a statistical significance of <0.05 (Ttest) between primary versus metastasis cell lines. The mass spectrometry proteomics data have been deposited to the ProteomeXchange Consortium via the PRIDE partner repository with the dataset identifier PXD060687.

*Western Blotting*

Cells were lysed using ice-cold RIPA lysis buffer (Millipore Sigma) with HALT protease and phosphatase inhibitors (Thermo Fisher). For western blot analysis, cell lysates or nucleolar fractions were resolved using SDS-PAGE and transferred to PVDF membrane. The membrane was blocked in 5% non-fat dry milk in Tris-buffered saline with 0.1% Tween-20 (TBST) and incubated with primary antibody overnight at 4°C. The membrane was washed in TBST and incubated with mouse or rabbit HRP-conjugated secondary antibody. The signal was visualized using ECL Prime (GE Healthcare, Pittsburgh, PA) and detected using the GE Amersham 600 Imager (GE Healthcare). The following antibodies were used: anti-α-tubulin−HRP (Cell Signaling), anti-Fibrillarin (Abcam), anti-S100A16 (Sigma), anti-E-cadherin (Cell Signaling), anti-Vimentin (Cell Signaling), anti-ZEB2 (Bethyl Labs, Montgomery, TX) anti-ZEB1 (Novus Biologicals, Centennial, CO).

*Real-time PCR*

RNA was isolated from cells using the RNeasy Mini Kit (Qiagen, Hilden, Germany). cDNA was generated using 1µg total RNA and High-Capacity cDNA kit (Thermo Fisher). Real-time PCR was performed using 40ng total cDNA per reaction, along with 2X TaqMan Fast Advance Master Mix (Thermo Fisher) and the following TaqMan primer probes: β-actin, CDH1, KRT14, SNAI1, VIM, ZEB1, ZEB2 (Thermo Fisher).

The steady state activity of RNA Pol I transcription was measured by monitoring levels of short-lived 5’ external transcribed spacer (5’ ETS) of the 47S pre-RNA by real-time PCR. Reactions were performed with 1:50 diluted cDNA with 2X Maxima SYBR Green Master Mix (Thermo Fisher) along with the following primer sets:

5’ETS 851-961 For- GAACGGTGGTGTGTCGTT Rev- GCGTCTCGTCTCGTCTCACT, Actin For- CATGTACGTTGCTATCCAGGC Rev-ctccttaatgtcacgcacgat.

Reactions were done in triplicate using Applied Biosystems StepOnePlus Real-time PCR machine. Analysis was done using ^ΔΔ^CT to determine relative fold changes in mRNA or 5’ETS transcripts.

*Immunohistochemistry*

Matched metastatic and primary breast carcinoma tissue microarrays (BRM961a, BRM961b, BR1008b, BR10010-L87, and BR20837a, TissueArray.com, Derwood, MD) were baked overnight at 60 °C. Slides were deparaffinized in a series of three xylene solutions for two minutes each, then rehydrated in 100%, 95%, and 70% ethanol solutions for two minutes each. Antigen unmasking was accomplished by sodium citrate antigen retrieval (12-minute boiling) and slides were allowed to cool in running water for 20 minutes. Slides were then placed in a deionized water bath and marked with a PapPen. Dual Endogenous Enzyme Block For Autostainer (Aligent, Santa Clara, CA) was added to tissue and allowed to incubate for 15 minutes at room temperature. Tissue was rinsed gently with Tris buffer and Tris buffer bath was briefly applied. Excess buffer was tapped off and tissues were blocked with 0.1% BSA in PBS for 40 minutes at room temperature. S100A16 antibody (Millipore Sigma), diluted 1:2500 in PBE buffer, was applied to tissues and incubated overnight in a humidified chamber at 4 °C. Primary antibody was rinsed off with Tris buffer and four Tris buffer baths were applied, five minutes each. Excess buffer was tapped off and EnVision+ System-HRP Labelled Polymer Anti-Rabbit (Aligent) was applied to tissue and incubated for 40 minutes at room temperature. Secondary was rinsed off with Tris buffer and four Tris buffer baths were applied, five minutes each. Tissue was incubated for 12 minutes with Liquid DAB + Substrate Chromogen System (Aligent) prepared according to manufacturer’s protocol. Excess was drained off and slides were placed in a deionized water bath for 1 minute. Counterstain was performed using filtered, undiluted Hematoxylin solution (Millipore Sigma) applied for three minutes. Slides were placed in a deionized water bath and placed under running tap water for four minutes. Slides were placed in 70%, 95%, then 100% ethanol solutions for two minutes each to dehydrate and then two minutes in a series of three xylene solutions. Epredia Cytoseal XYL (Fisher Scientific, Hampton, NH) was used to mount coverslips. Images were captured with the Leica Aperio ScanScope CS pathology slide scanner and immunoreactive scoring was assigned. The immunoreactive scores from final analysis ensures that each case was represented only once. Immunoreactive scores between the primary breast carcinomas and lymph node metastases were compared and significance was analyzed using a t-test.

*Publicly Available Data Set Analyses*

Survival data was obtained from Kaplan Meier Plotter ([Kaplan-Meier plotter (kmplot.com)](https://kmplot.com/analysis/)) mRNA gene chip data set. Patients were split by trichotomization of S100A16 expression and tertile 1 vs tertile 3 relapse-free survival (RFS) was compared. To determine significance, log-rank p-value was obtained with a Mantel-Cox test.

The data of “Differentiation dynamics of the developing mammary gland revealed by single-cell RNA-sequencing” were interactively browsed for genes of interest using publicly available database at https://marionilab.cruk.cam.ac.uk/mammaryGland/.

To determine if expression of S100A16 in the primary tumor is correlated to events of metastasis, the Sweden Canceromics Analysis Network – Breast (SCAN-B) data set was downloaded from Gene Expression Omnibus and is available under accession GSE60788. Samples were parsed by lymph node metastases positive or lymph node metastases negative patients and S100A16 expression from bulk RNA-seq data was queried. A t-test was used to obtain a p-value to determine significance. Additionally, samples were divided into quartiles of top 25% and bottom 25% based on S100A16 expression and denoted as ‘high’ and ‘low’. Gene set enrichment analysis (GSEA) was performed utilizing GSEA v4.2.3 Mac App with a collection of MSigDB annotated gene sets (UC San Diego and Broad Institute).

The AURORA data set was downloaded from Gene Expression Omnibus under accession number GSE193103, comprising patient samples from primary (n=44) and metastatic tumors (n=79). Samples were further stratified based on location of metastasis (bone n=2 lung n=8 brain n=9 and lymph n=11). GSEA was performed utilizing GSEA v4.2.3 Mac App with a collection of MSigDB annotated gene sets (UC San Diego and Broad Institute). Annotations from GSEA were illustrated as bubble plots using the “ggplot2” package in RStudio v4.1.2 (Boston, MA, USA).

*In vivo Tumor Models*

Luciferase-GFP expressing MCF10CA1acl.1 non-silenced or shS100A16 were suspended in HBSS (Thermo Fisher) and 1.5 x 10^6^ cells were injected into the inguinal mammary fat pad of 7-week-old female athymic nude mice. Tumor growth was monitored with caliper readings three times a week. Once tumors reached a mean tumor diameter of 10mm^2^ mice were anesthetized and tumors were sterile resected. Mice were monitored for lung metastasis via bioluminescent imaging with a IVIS Lumina III (Perkin Elmer, Waltham, MA) to detect the presence of GFP labeled cells.

All animal studies were conducted in accordance with, and with the approval of, the Institutional Animal Care and Use Committee (IACUC) of University of Alabama at Birmingham (UAB). As per approved IACUC guidelines tumors were resected upon a mean tumor diameter no larger than 10mm^2^; however, to maintain experimental time line integrity one tumor was allowed to grow larger than the prescribed 10mm^2^. Raw tumor growth data has been provided in Supplemental Table 3.

*AgNOR Staining*

Slides were deparaffinized and rehydrated before incubation in the staining solution (one-part 2% gelatin, 1% formic acid solution, and two parts 50% silver nitrate in water, at room temperature). Images were taken using Nikon Eclipse Ti (Nikon Instruments Inc.) at 90X. In total, nucleoli were quantified across 25 random fields from multiple tumors with at least 40 cells counted per field. Results were represented as the percentage of cells containing 1, 2, or 3+ nucleoli per cell.

*In Situ Hybridization and Quantitative Image Analysis*

Tissue sections were processed using the RNAscope Multiplex Fluorescent v2 Assay with the RNAscope Probe (ACD Bio, Newark, CA). Briefly, slides were baked at 60°C for 1 hour, deparaffinized in two fresh xylene baths (5 minutes each) and two subsequent immersions in 100% ethanol (2 minutes each) and then air-dried. Endogenous peroxidase activity was quenched with RNAscope Hydrogen Peroxide (applied for 10 minutes), followed by thorough rinsing in distilled water. Antigen retrieval was performed by incubating the slides in a preheated 1X Target Retrieval Reagent (≥99°C) in a steamer for 15 minutes after a brief acclimation in distilled water, then briefly rinsed and dehydrated with ethanol before drying. A hydrophobic barrier was drawn around each section, and protease digestion was carried out using RNAscope Protease Plus in a HybEZ Humidity Control Tray at 40°C for 30 minutes.

For probe hybridization, a pre-warmed C1 RNA45S probe was applied (approximately 4 drops per 0.75” × 0.75” area) and incubated at 40°C for 2 hours. The unbound probe was removed by washing twice in pre-warmed 1X Wash Buffer. Signal amplification proceeded through sequential incubations with AMP 1 (30 minutes), AMP 2 (30 minutes), and AMP 3 (15 minutes) at 40°C, each followed by two 2-minute washes in 1X Wash Buffer. For signal development, the HRP reagent specific to the C1 channel was applied for 15 minutes at 40°C, followed by TSA Vivid Dye 570 (diluted 1:1500) for 30 minutes at 40°C. An HRP blocking step (15 minutes at 40°C) stabilized the fluorescent signal. Finally, nuclei were counterstained with DAPI (applied for 30 seconds), and slides were mounted using ProLong Gold Antifade Mountant under a coverslip. Fluorescent images were acquired using a Nikon Eclipse Ti-U microscope.

A custom ImageJ macro was developed to quantify particle counts in the resulting TIFF images. For each image, the macro determined the image dimensions and, when only one channel was present, treated each slice as a separate channel. Each channel was processed by duplicating the active slice and converting it to 8-bit. A fixed threshold (35–255) was applied to create a binary mask, and holes were filled before applying watershed segmentation to separate adjacent particles. Particle quantification was then performed using the “Analyze Particles” function with size limits set from 1 to 10,000 pixels and a circularity range of 0.50–1.00. The resulting particle counts for each channel were recorded, and in downstream analysis, the counts obtained from the TRITC (RNAscope) channel were divided by those from the DAPI channel to yield a normalized metric.

*Pulmonary Metastasis Assays*

Pulmonary metastasis assays (PuMA) were carried out adopting the protocol published by Mendoza et al. 2x10^5^GFP-expressing MCF10CA1acl.1 Non-silenced or shS100A16 cells were injected into the tail vein of athymic nude mice. Fifteen minutes post injection, mice were humanely euthanized, and lungs were cannulated with 0.6% agarose in assay media (M-199 supplemented with 1.0 μg/mL crystalline bovine insulin, 0.1 μg/mL hydrocortisone, 0.1 μg/mL retinyl acetate, 100 U/mL penicillin and 100 μg/mL streptomycin, and 7.5% sodium bicarbonate). Lung sections were placed on a 2 x 2 x 0.7 cm piece of Surgifoam (Ethicon, Somerville, NJ) soaked in culture media. Lung sections were flipped over with each media change twice weekly.

Pictures were captured using a Nikon SMZ800 stereo zoom microscope. Images were analyzed using ImageJ-FIJI software and area corrected total cell fluorescence (CTCF) was calculated as CTCF = Integrated Density – (Area of selected cell X Mean fluorescence of background readings).
